# Supplementary material for: The effects of the E3 ubiquitin–protein ligase UBR7 of Frankliniella occidentalis on the ability of insects to acquire and transmit TSWV
Source: PeerJ. 2023 May 9;11:e15385. doi: 10.7717/peerj.15385 (PMC10178284; doi:10.7717/peerj.15385)
Supplement: Supplemental Information 1 [file peerj-11-15385-s001.docx]

**Experimental records**

**Contents**

[1. Bioinformatic analysis 3](#_Toc127432189)

[1.1 Transcriptome data download information 3](#_Toc127432190)

[1.2 Results of transcriptome analysis 6](#_Toc127432192)

[1.3 Prediction analysis of proteins 10](#_Toc127432194)

[1.4 Homologous alignment of amino acid sequence 11](#_Toc127432196)

[2. Full length verification 12](#_Toc127432198)

[2.1 Sequencing results after TA cloning 12](#_Toc127432199)

[2.2 Putative translated protein sequences 12](#_Toc127432200)

[3. Phylogenetic tree related data 14](#_Toc127432201)

[4. Antibody preparation 22](#_Toc127432203)

[4.1 Project Information 22](#_Toc127432204)

[4.2 Product Information 22](#_Toc127432205)

[4.3 QC (Quality Control) Results 22](#_Toc127432207)

[5. RT-qPCR standard curve preparation 23](#_Toc127432209)

[5.1. Standard preparation 23](#_Toc127432210)

[5.2. Standard curve drawing 24](#_Toc127432211)

[6. Surface plasmon resonance related data 25](#_Toc127432213)

[6.1. Prokaryotic expression 25](#_Toc127432214)

[6.2 L scale-up purification 27](#_Toc127432217)

[6.3. Surface plasmon resonance 28](#_Toc127432219)

[6.4 Protein mass spectrometry 30](#_Toc127432223)

[7. GST pull down related data 37](#_Toc127432230)

[7.1. Prokaryotic expression 38](#_Toc127432231)

[8. Amino acid sequence alignment of "hemocyanin subunit 1 precursor" 39](#_Toc127432230)

[8.1. Amino acid sequence alignment 39](#_Toc127432231)

[8.2. Amino acid sequence information for “hemocyanin subunit type 1 precursor” sequence alignment 39](#_Toc127432231)

**Contents**

[Supplementary table 1：Transcriptome information of *Frankliniella occidentalis* from NCBI SPR 3](#_Toc127108677)

[Supplementary table 2：The top twenty most differentially-abundant transcript sequences in three developmental stages of *Frankliniella occidentalis* in response to tomato spotted wilt virus (TSWV) infection 6](#_Toc127108679)

[Supplementary table 3: Bioinformatics prediction analysis of physicochemical properties of proteins 10](#_Toc127108681)

[Supplementary table 4: Information about antibody preparation products 22](#_Toc127108692)

[Supplementary table 5: Quality Control of antibody preparation products 22](#_Toc127108694)

[Supplementary table 6: Data related to standard curve 24](#_Toc127108698)

[Supplementary table 7: Experimental parameters of SPR 28](#_Toc127108706)

[Supplementary table 8: Peptides interacting with UBR7-Domino identified by SPR and LC-MS/MS 30](#_Toc127108710)

[Supplementary table 9: The information of protein mass spectrometry 31](#_Toc127108711)

[Supplementary table 10. Search parameters of SPR 31](#_Toc127108712)

[Supplementary table 11: Decoy search summary of SPR 32](#_Toc127108713)

[Supplementary figure 1. Protocol for prokaryotic expression 26](#_Toc127108701)

[Supplementary figure 2. Expression tests of the target protein UBR7-domino 26](#_Toc127108702)

[Supplementary figure 3. Reducing-PAGE analysis. Final sample QC. 2µg of sample loaded 27](#_Toc127108704)

[Supplementary figure 4. Sample signal diagram 28](#_Toc127108707)

[Supplementary figure 5. Sample elution 29](#_Toc127108708)

[Supplementary figure 6. Secondary mass spectrogram 32](#_Toc127108714)

[Supplementary figure 7. Mascot search result 37](#_Toc127108715)

[Supplementary figure 8. Amino acid sequence alignment of "hemocyanin subunit 1 precursor" 39](#_Toc127108715)

[Supplementary txt 1. Amino-acid sequences used for homologous alignment 11](#_Toc127108683)

[Supplementary txt 2. Amino-acid sequences used for phylogenetic tree 14](#_Toc127108688)

# 1. Bioinformatic analysis

### 1.1 Transcriptome data download information

## Supplementary table 1：Transcriptome information of *Frankliniella occidentalis* from NCBI SPR

**Table S1：Transcriptome information of *Frankliniella occidentalis* from NCBI SPR**

| **Group Name** | **Original name** | **Tissue** | **Existence of TSWV** | **Description** | **Instar** | **Accession number** | | **Download URL** |
| --- | --- | --- | --- | --- | --- | --- | --- | --- |
|  |  |  |  |  |  | **SRR** | **SPX** |  |
| V-HL | NV_L1_1 | whole body | V- | No Virus-first instar larvae (L1) Biorep 1 | First instar larvae (L1) | SRR7085636 | SRX4015378 | ftp://ftp-trace.ncbi.nih.gov/sra/sra-instant/reads/ByRun/sra/SRR/SRR708/SRR7085636/SRR7085636.sra |
|  | NV L1-21 Rep2 | whole body | V- | No Virus-first instar larvae (L1) Biorep 2 | First instar larvae (L1) | SRR7140918 | SRX4061765 | ftp://ftp-trace.ncbi.nih.gov/sra/sra-instant/reads/ByRun/sra/SRR/SRR714/SRR7140918/SRR7140918.sra |
|  | NV L1-21 Rep3 | whole body | V- | No Virus-first instar larvae (L1) Biorep 3 | First instar larvae (L1) | SRR7140917 | SRX4061766 | ftp://ftp-trace.ncbi.nih.gov/sra/sra-instant/reads/ByRun/sra/SRR/SRR714/SRR7140917/SRR7140917.sra |
| V-HP | NV P1 Rep1 | whole body | V- | No Virus-prepupae (P1) Biorep 1 | Prepupae (P1) | SRR7255053 | SRX4159448 | ftp://ftp-trace.ncbi.nih.gov/sra/sra-instant/reads/ByRun/sra/SRR/SRR725/SRR7255053/SRR7255053.sra |
|  | NV P1 Rep2 | whole body | V- | No Virus-prepupae (P1) Biorep 2 | Prepupae (P1) | SRR7255052 | SRX4159449 | ftp://ftp-trace.ncbi.nih.gov/sra/sra-instant/reads/ByRun/sra/SRR/SRR725/SRR7255052/SRR7255052.sra |
|  | NV P1 Rep3 | whole body | V- | No Virus-prepupae (P1) Biorep 3 | Prepupae (P1) | SRR7255051 | SRX4159450 | ftp://ftp-trace.ncbi.nih.gov/sra/sra-instant/reads/ByRun/sra/SRR/SRR725/SRR7255051/SRR7255051.sra |

Continued：

| V-HA | NV Ad-24 Rep1 | whole body | V- | No Virus-adult (Ad) Biorep 1 | Adult (mixed females and males) | SRR7255050 | SRX4159451 | ftp://ftp-trace.ncbi.nih.gov/sra/sra-instant/reads/ByRun/sra/SRR/SRR725/SRR7255050/SRR7255050.sra |
| --- | --- | --- | --- | --- | --- | --- | --- | --- |
|  | NV Ad-24 Rep2 | whole body | V- | No Virus-adult (Ad) Biorep 2 | Adult (mixed females and males) | SRR7255049 | SRX4159452 | ftp://ftp-trace.ncbi.nih.gov/sra/sra-instant/reads/ByRun/sra/SRR/SRR725/SRR7255049/SRR7255049.sra |
|  | NV Ad-24 Rep3 | whole body | V- | No Virus-adult (Ad) Biorep 3 | Adult (mixed females and males) | SRR7255048 | SRX4159453 | ftp://ftp-trace.ncbi.nih.gov/sra/sra-instant/reads/ByRun/sra/SRR/SRR725/SRR7255048/SRR7255048.sra |
| V+HL | V L1-21 Rep1 | whole body | V+ | TSWV-first instar larvae (L1) Biorep 1 | First instar larvae (L1) | SRR7255047 | SRX4159454 | ftp://ftp-trace.ncbi.nih.gov/sra/sra-instant/reads/ByRun/sra/SRR/SRR725/SRR7255047/SRR7255047.sra |
|  | V L1-21 Rep2 | whole body | V+ | TSWV-first instar larvae (L1) Biorep 2 | First instar larvae (L1) | SRR7255046 | SRX4159455 | ftp://ftp-trace.ncbi.nih.gov/sra/sra-instant/reads/ByRun/sra/SRR/SRR725/SRR7255046/SRR7255046.sra |
|  | V L1-21 Rep3 | whole body | V+ | TSWV-first instar larvae (L1) Biorep 3 | First instar larvae (L1) | SRR7255055 | SRX4159446 | ftp://ftp-trace.ncbi.nih.gov/sra/sra-instant/reads/ByRun/sra/SRR/SRR725/SRR7255055/SRR7255055.sra |

Continued：

| V+HP | V P1 Rep1 | whole body | V+ | TSWV-prepupae (P1) Biorep 1 | Prepupae (P1) | SRR7255054 | SRX4159447 | ftp://ftp-trace.ncbi.nih.gov/sra/sra-instant/reads/ByRun/sra/SRR/SRR725/SRR7255054/SRR7255054.sra |
| --- | --- | --- | --- | --- | --- | --- | --- | --- |
|  | V P1 Rep2 | whole body | V+ | TSWV-prepupae (P1) Biorep 2 | Prepupae (P1) | SRR7255058 | SRX4159443 | ftp://ftp-trace.ncbi.nih.gov/sra/sra-instant/reads/ByRun/sra/SRR/SRR725/SRR7255058/SRR7255058.sra |
|  | V P1 Rep3 | whole body | V+ | TSWV-prepupae (P1) Biorep 3 | Prepupae (P1) | SRR7255057 | SRX4159444 | ftp://ftp-trace.ncbi.nih.gov/sra/sra-instant/reads/ByRun/sra/SRR/SRR725/SRR7255057/SRR7255057.sra |
| V+HA | V Ad-24 Rep1 | whole body | V+ | TSWV-adult (Ad) Biorep 1 | Adult (mixed females and males) | SRR7255060 | SRX4159441 | ftp://ftp-trace.ncbi.nih.gov/sra/sra-instant/reads/ByRun/sra/SRR/SRR725/SRR7255060/SRR7255060.sra |
|  | V Ad-24 Rep2 | whole body | V+ | TSWV-adult (Ad) Biorep 2 | Adult (mixed females and males) | SRR7255059 | SRX4159442 | ftp://ftp-trace.ncbi.nih.gov/sra/sra-instant/reads/ByRun/sra/SRR/SRR725/SRR7255059/SRR7255059.sra |
|  | V Ad-24 Rep3 | whole body | V+ | TSWV-adult (Ad) Biorep 3 | Adult (mixed females and males) | SRR7255056 | SRX4159445 | ftp://ftp-trace.ncbi.nih.gov/sra/sra-instant/reads/ByRun/sra/SRR/SRR725/SRR7255056/SRR7255056.sra |

### 1.2 Results of transcriptome analysis

## Supplementary table 2：The top twenty most differentially-abundant transcript sequences in three developmental stages of *Frankliniella occidentalis* in response to tomato spotted wilt virus (TSWV) infection

**Table S2: The top twenty most differentially-abundant transcript sequences in three developmental stages of *Frankliniella occidentalis* in response to tomato spotted wilt virus (TSWV) infection.**

|  | **Gene ID** | **Stage-time** | **Fold-change** | **Direction** | **Description** | **Length** | **e-Value** | **GO Names list** | **InterPro domain present** |
| --- | --- | --- | --- | --- | --- | --- | --- | --- | --- |
| 1 | TCONS_00003267 | Pupae | 17.86 | UP | NA | 464 | - | - | YES |
| 2 | FOCC017110-RA | Adults | 15.38 | UP | NA | 1089 | - | - | YES |
| 3 | FOCC003013-RA | Adults | 13.31 | UP | hemocyanin subunit type 1 precursor or hexamerin | 2386 | 1.1E-166 | F:metal ion binding; P:oxidation-reduction process; F:oxidoreductase activity; P:metabolic process; F:monophenol monooxygenase activity | YES |
| 4 | XLOC_000373 | Larvae | 9.44 | DOWN | NA | 876 | - | - | NO |
| 5 | FOCC012829-RA | Adults | 8.87 | UP | arylphorin subunit alpha | 2892 | 3.9E-168 | C:extracellular space; F:nutrient reservoir activity; C:extracellular region | YES |

continue：

|  | **Gene ID** | **Stage-time** | **Fold-change** | **Direction** | **Description** | **Length** | **e-Value** | **GO Names list** | **InterPro domain present** |
| --- | --- | --- | --- | --- | --- | --- | --- | --- | --- |
| 6 | FOCC004760-RA | Adults | 8.64 | UP | hemocyanin subunit type 1 precursor or hexamerin | 1552 | 2.2E-110 | P:oxidation-reduction process; F:monophenol monooxygenase activity; F:oxidoreductase activity; F:metal ion binding; P:metabolic process; C:extracellular space; F:nutrient reservoir activity; C:extracellular region | NO |
| 7 | TCONS_00001377 | Adults | 8.61 | UP | NA | 682 | - | - | NO |
| 8 | FOCC004194 | Larvae | 8.22 | DOWN | ejaculatory bulb-specific protein 3 | 571 | 2.3E-41 | - | NO |
| 9 | TCONS_00012780 | Pupae | 8.21 | UP | NA | 844 | - | - | NO |
| 10 | XLOC_013044 | Pupae | 7.85 | DOWN | NA | 614 | - | - | NO |
| 11 | FOCC001757 | Larvae | 7.15 | DOWN | NA | 1865 | - | - | YES |

continue：

|  | **Gene ID** | **Stage-time** | **Fold-change** | **Direction** | **Description** | **Length** | **e-Value** | **GO Names list** | **InterPro domain present** |
| --- | --- | --- | --- | --- | --- | --- | --- | --- | --- |
| 12 | XLOC_015448 | Adults | 6.21 | UP | arylphorin subunit c223-like | 520 | 5.3E-08 | F:metal ion binding; P:oxidation-reduction process; F:oxidoreductase activity; P:metabolic process; F:nutrient reservoir activity | NO |
| 13 | FOCC016855-RA | Adults | 6.14 | UP | hemocyanin subunit type 1 precursor or hexamerin | 901 | 3.9E-60 | F:metal ion binding; P:oxidation-reduction process; F:oxidoreductase activity; P:metabolic process | NO |
| 14 | FOCC009158-RA | Pupae | 5.78 | UP | NA | 1806 | - | - | YES |
| 15 | XLOC_015687 | Adults | 5.58 | DOWN | NA | 704 | - | - | NO |
| 16 | FOCC016444-RA | Pupae | 5.3 | UP | NA | 1015 | - | - | NO |
| 17 | FOCC015675 | Larvae | 5.27 | DOWN | NA | 847 | - | - | NO |
| 18 | CUFF | Pupae | 5.25 | UP | glucose dehydrogenase | 3772 | 1.1E-88 | P:oxidation-reduction process; F:oxidoreductase activity, acting on CH-OH group of donors; F:flavin adenine dinucleotide binding | YES |

continue：

|  | **Gene ID** | **Stage-time** | **Fold-change** | **Direction** | **Description** | **Length** | **e-Value** | **GO Names list** | **InterPro domain present** |
| --- | --- | --- | --- | --- | --- | --- | --- | --- | --- |
| 19 | FOCC006231 | Larvae | 5.04 | DOWN | trypsin 1 | 1149 | 8E-56 | - | NO |
| 20 | FOCC013480 | Larvae | 4.93 | DOWN | alpha-tocopherol transfer isoform x2 | 1983 | 1.1E-97 | - | YES |

"FOCC (transcript assembled to Maker-predicted gene models), CUFF (assembled transcript in close proximity or partially overlaps Maker-predicted gene model, i.e., isoforms or variants), TCONS (novel contig (transcript fragment) on scaffold with no Maker-predicted gene model). NA, non-annotated by Blastx"

CUFFDIFF output for differentially-expressed transcripts in response to virus (TSWV to healthy) for the three *F. occidentalis* developmental stage-times and levels of significance after Benjamini-Hochberg correction for multiple tests (FDR = 0.05, q-value < 0.05)

Gene descriptions, length, and e-values of matches are provided; NA = no significant Blastx match with known proteins at E < 10^-3^

### 1.3 Prediction analysis of proteins

## Supplementary table 3: Bioinformatics prediction analysis of physicochemical properties of proteins

|  | | | | | | | | | | | | |
| --- | --- | --- | --- | --- | --- | --- | --- | --- | --- | --- | --- | --- |
| **Table S3. Bioinformatics prediction analysis of physicochemical properties of proteins** | | | | | | | | | | | | |
| **Name** | **Formula** | **Total number of atoms** | **Gene ID** | **ORF (bp)** | **Number of amino acids** | **Molecular weight** | **Theoretical pI** | **Asp + Glu** | **Arg + Lys** | **Instability index** | **Aliphatic index** | **Grand average of hydropathicity** |
| UBR7 | C_2007_H_3108_N_558_O_662_S_30_ | 6365 | XM_026422690.1 | 1246 | 414 | 46551.9 | 4.89 | 66 | 44 | 50.46 | 60.77 | -0.714 |

### 1.4 Homologous alignment of amino acid sequence

## Supplementary txt 1. Amino-acid sequences used for homologous alignment

>UBR7-domino

LNCTYPQGYVKRQALYACITCIPAGSDQNRGGVCLACSYSCHENHDLVELYTKRNFRCDCGNSQFGSNKCNLEPVKEVNEKNKYNQNFKGVYCTCKRPYPDPEDTNDDEMIQCIICEDWYHGRHLGVNKAIPKDYGEMVCEQC

>hypothetical protein EJD97_012353 [Solanum chilense] TMW92961.1

KECTYSKGYMKRQAIFSCLTCTPDGNAGVCTACSLSCHDGHEILELWTKRNFRCDCGNSKFGEFFCKLDASKDVENTKNSYNHNFKGSYCTCGRPYPDPDVEDQLENLQCCICEDWFHEEHLGLESSNMVPRDDKGEPQFEDLICQGC

>putative E3 ubiquitin-protein ligase UBR7 [Solanum lycopersicum] XP_004245515.1

MADAFEEDGENTVSIGEFLQDLEEQELEAGLVLGGDEGKECTYSKGYMKRQAIFSCLTCTPDGIAGVCTACSLSCHDGHEILELWTKRNFRCDCGNSKFGEFFCKLDASKDVENTKNSYNHNFKGSYCTCGRPYPDPDVEDQLENLQCCICEDWFHEEHLGLESSDMVPRDDKGEPQFEDLICQGC

>putative E3 ubiquitin-protein ligase UBR7 [Solanum pennellii] XP_015085857.1

KECTYSKGYMKRQAIFSCLTCTPDGNAGVCTACSLSCHDGHEILELWTKRNFRCDCGNSKFGEFFCKLDASKDVENTKNSYNHNFKGSYCTCGRPYPDPDVEDQLENLQCCICEDWFHEEHLGLESSNMVPRDDKGEPQFEDLICQGC

>PREDICTED: putative E3 ubiquitin-protein ligase UBR7 [Solanum tuberosum] XP_006343859.1

KECTYSKGYMKRQAIFSCLTCTPNGNAGVCTACSLSCHDGHEILELWTKRNFRCDCGNSKFGEFFCKLDASKDVENTKNSYNHNFKGSYCTCGRPYPDPDVEDQLENLQCCICEDWFHEEHLGLESSDMVPRDDKGEPQFEDLICQGC

>PREDICTED: putative E3 ubiquitin-protein ligase UBR7 [Nicotiana attenuata] XP_019226065.1

KECTYSKGYMKRQAIFSCLTCTPDGNAGVCTACSLSCHDGHEILELWTKRNFRCDCGNSKFGEFFCKLDASKDVENTENSYNHNFKGSYCTCCRPYPDPDVEDQLENLQCCICEDWFHEEHLGLESSDMVPRDENGEPQFEDLICQGC

>PREDICTED: putative E3 ubiquitin-protein ligase UBR7 [Nicotiana sylvestris] XP_009766510.1

KECTYSKGYMKRQAIFSCLTCTPDGNAGVCTACSLSCHDGHEILELWTKRNFRCDCGNSKFGEFFCKLDASKDVENTKNSYNHNFKGSYCTCCRPYPDPDVEDQLENLQCCICEDWFHEEHLGLESSDMIPRDENGEPLFEDLICQGC

>putative E3 ubiquitin-protein ligase UBR7 [Nicotiana tomentosiformis] XP_009601236.1

KECTYSKGYMKRQAIFSCLTCTPDGNAGVCTACSLSCHDGHEILELWTKRNFRCDCGNSKFGEFFRKLDASKDVENTENSYNHNFKGSYCTCCRPYPDPDVEDQLENLQWCICEDWFHEEHLGLESSDMVPRDENGEPQFEDLICQGC

>PREDICTED: putative E3 ubiquitin-protein ligase UBR7 [Nicotiana tabacum] XP_016506890.1

KECTYSKGYMKRQAIFSCLTCTPDGNAGVCTACSLSCHDGHEILELWTKRNFRCDCGNSKFGEFFCKLDASKDVENTENSYNHNFKGSYCTCCRPYPDPDVEDQLENLQCCICEDWFHEEHLGLESSDMVPRDENGEPQFEDLICQGC

>hypothetical protein HAX54_040930 [Datura stramonium] MCD7459458.1

KECTYSKGYMKRQAIFSCLTCTPDGNAGVCTACSLSCHDGHEILELWTKRNFRCDCGNSKFGEFFCKLDASKDVENTKNLYNHNFKGSYCTCGRPYPDPDVEDQLENLQCCICEDWFHEEHLGLESSDMVPRDEKGEPQFEDLICQGC

# 2. Full length verification

### 2.1 Sequencing results after TA cloning

ATGGCTGAAAAATCTTCAAGTGTTGAACCTCCGGTCAATGGTCAGGAAGCAGATGATGACGAAAGTAACGCTATTACTATGGTGGATGTTTTAAAAGAGGAGACTGAATTGGAAGAGGATGCCAATGCGGTGTTGGGTGGATCTGATCCCCTAAATTGCACTTACCCTCAGGGCTATGTGAAGCGACAAGCTCTTTATGCTTGTATTACATGCATTCCAGCTGGCTCTGACCAAAATAGAGGTGGTGTATGCCTTGCTTGCAGTTACAGCTGCCATGAGAATCACGATCTTGTAGAGCTGTACACCAAGAGGAATTTTCGCTGTGATTGTGGCAACTCACAATTTGGCAGCAACAAGTGCAACTTGGAGCCAGTGAAAGAAGTAAATGAAAAGAACAAGTACAATCAAAATTTTAAAGGAGTGTACTGCACTTGCAAGAGACCATATCCTGATCCAGAAGACACCAATGATGATGAAATGATCCAATGTATAATTTGTGAAGACTGGTATCATGGCAGGCACTTGGGAGTTAACAAAGCAATTCCAAAAGATTACGGCGAAATGGTCTGTGAACAGTGCATGAGCAAACATTCGTTTTTGTGGAACTATGCTGGATTGTGTTTGACTAAAGCAACTTCTTCTGATCAAGAAGTAGAAGTGGATCAGGATCCCTCTACTTTCAAACAAACCTCTGCTTCAGATGATGTAGAGCCATCCACACCCACAATTCCTATCAATGGTTCAGCAGGAGTTGATTTCAACACCCCTGGCTCCTCATCCCAAAAGAGCAATATTGAAACACCTATTCATGATGGTAAAGAATGTATTTTGATGAACTTTAAACCAGTTGAACAACTTAAGGGAGCAACATTTTGGCCTGAAGGCTGGCGCAAACAGTTGTGTCTTTGCATCAAGTGCCTGGAAAATTATGAAGCTAATGGGGTTTCCTTTCTTACTGATTTGCAAGATACTGTTCAGTATTATGAAGAACAGGGAAAGGCTAAAGCAGCAAATGGACAAGCGAGTTCTCAGTATGATCATGCAATGCATGCCTTGTCACAGTTAGATCGCACTGCTCAAATTGAAGCTATACATGGCTACAATGACATGAAAGACCAGCTGAAGGAATACTTGCAGAAGTTTGCAGAGAACAGGAAGGTTGTACGAGAGGAAGATATTCGCGAGTTTTTCAGTCAAATGGCTCGTAAGCGTCCTCGTGTTGAGGTTTCATCATTTTGTCGTTAG

### 2.2 Putative translated protein sequences

MAEKSSSVEPPVNGQEADDDESNAITMVDVLKEETELEEDANAVLGGSDPLNCTYPQGYVKRQALYACITCIPAGSDQNRGGVCLACSYSCHENHDLVELYTKRNFRCDCGNSQFGSNKCNLEPVKEVNEKNKYNQNFKGVYCTCKRPYPDPEDTNDDEMIQCIICEDWYHGRHLGVNKAIPKDYGEMVCEQCMSKHSFLWNYAGLCLTKATSSDQEVEVDQDPSTFKQTSASDDVEPSTPTIPINGSAGVDFNTPGSSSQKSNIETPIHDGKECILMNFKPVEQLKGATFWPEGWRKQLCLCIKCLENYEANGVSFLTDLQDTVQYYEEQGKAKAANGQASSQYDHAMHALSQLDRTAQIEAIHGYNDMKDQLKEYLQKFAENRKVVREEDIREFFSQMARKRPRVEVSSFCR*

# 3. Phylogenetic tree related data

## Supplementary txt 2. Amino-acid sequences used for phylogenetic tree

>▲Target protein UBR7 [Frankliniella occidentalis]

MAEKSSSVEPPVNGQEADDDESNAITMVDVLKEETELEEDANAVLGGSDPLNCTYPQGYVKRQALYACITCIPAGSDQNRGGVCLACSYSCHENHDLVELYTKRNFRCDCGNSQFGSNKCNLEPVKEVNEKNKYNQNFKGVYCTCKRPYPDPEDTNDDEMIQCIICEDWYHGRHLGVNKAIPKDYGEMVCEQCMSKHSFLWNYAGLCLTKATSSDQEVEVDQDPSTFKQTSASDDVEPSTPTIPINGSAGVDFNTPGSSSQKSNIETPIHDGKECILMNFKPVEQLKGATFWPEGWRKQLCLCIKCLENYEANGVSFLTDLQDTVQYYEEQGKAKAANGQASSQYDHAMHALSQLDRTAQIEAIHGYNDMKDQLKEYLQKFAENRKVVREEDIREFFSQMARKRPRVEVSSFCR

>gi|1770339816|ref|XP_031339240.1| putative E3 ubiquitin-protein ligase UBR7 [Photinus pyralis]

MSEENNTTLNETVEDPEQSIVTLGEVLQSEQEMIDDVNAVLGAIDDKKCTYSEGYMKRQALYSCLTCIPEAKSDITKAAGVCLGCCFHCHEGHELVELYTKRNFRCDCGNTKFANKSCKLETTKTDANEFNEYNQNFCGTYCSCNRPYPDPEDSVEDEMIQCILCEDWYHTRHLGVEFPRNVSYAEMCCESCIKQHPFLLYYGALCVKAADPNAEAESKEDEKDDNEVDVAQTDEKTDKETCTKPKDKSGLVTTKFWTDIEWRKQLCTCPECIQIYEEEKVMFLLDPQDTVHVYEEKGKAKAREFEEKQQENGMKFLNSLSRVPLMEALAGYNDMKQHLIEYLKKFAENKKVVREEDIHEFFSNYDAKKKQKSEVPHFCG

>gi|1770339059|ref|XP_031338892.1| putative E3 ubiquitin-protein ligase UBR7 [Photinus pyralis]

MSEENNTTLNETVEDPEQSIVTLGEVLQSEQEMIDDVNAVLGAIDDKKCTYSEGYMKRQALYSCLTCIPEAKSDITKAAGVCLGCCFHCHEGHELVELYTKRNFRCDCGNTKFANKSCKLETTKTDANEFNEYNQNFCGTYCSCNRPYPDPEDSVEDEMIQCILCEDWYHTRHLGVEFPRNVSYAEMCCESCIKQHPFLLYYGALCVKAADPNAEAESKEDEKDDNEVDVAQTDEKTDKETCTKPKDKSGLVTTKFWTDIEWRKQLCTCPECIQIYEEEKVMFLLDPQDTVHVYEEKGKAKAREFEEKQQENGMKFLNSLSRVPLMEALAGYNDMKQHLIEYLKKFAENKKVVREEDIHEFFSNYDAKKKQKSEVPHFCG

>gi|1755129319|gb|KAB0799755.1| hypothetical protein PPYR_07635 [Photinus pyralis]

MSEENNTTLNETVEDPEQSIVTLGEVLQSEQEMIDDVNAVLGAIDDKKCTYSEGYMKRQALYSCLTCIPEAKSDITKAAGVCLGCCFHCHEGHELVELYTKRNFRCDCGNTKFANKSCKLETTKTDANEFNEYNQNFCGTYCSCNRPYPDPEDSVEDEMIQCILCEDWYHTRHLGVEFPRNVSYAEMCCESCIKQHPFLLYYGALCVKAADPNAEAESKEDEKDDNEVDVAQTDEKTDKETCTKPKDKSGLVTTKFWTDIEWRKQLCTCPECIQIYEEEKVMFLLDPQDTVHVYEEKGKAKAREFEEKQQENGMKFLNSLSRVPLMEALAGYNDMKQHLIEYLKKFAENKKVVREEDIHEFFSNYDAKKKQKSEVPHFCG

>gi|1755129326|gb|KAB0799762.1| hypothetical protein PPYR_07642 [Photinus pyralis]

MSEENNTTLNETVEDPEQSIVTLGEVLQSEQEMIDDVNAVLGAIDDKKCTYSEGYMKRQALYSCLTCIPEAKSDITKAAGVCLGCCFHCHEGHELVELYTKRNFRCDCGNTKFANKSCKLETTKTDANEFNEYNQNFCGTYCSCNRPYPDPEDSVEDEMIQCILCEDWYHTRHLGVEFPRNVSYAEMCCESCIKQHPFLLYYGALCVKAADPNAEAESKEDEKDDNEVDVAQTDEKTDKETCTKPKDKSGLVTTKFWTDIEWRKQLCTCPECIQIYEEEKVMFLLDPQDTVHVYEEKGKAKAREFEEKQQENGMKFLNSLSRVPLMEALAGYNDMKQHLIEYLKKFAENKKVVREEDIHEFFSNYDAKKKQKSEVPHFCG

>gi|1812417589|gb|KAF2882349.1| hypothetical protein ILUMI_23833 [Ignelater luminosus]

MSEEHDTSMDQTVDEGDQEVVTLKDILQSEQELMDDANAVLGAIDDKKCTYSEGYMKRQALYSCLTCVPEARTDVSKAAGVCLACCLHCHEGHELIELYTKRNFRCDCGNKKFSNLTCILDNVKSSINELNQYNQNFSGVYCTCSRPYPDPEDPIEDDMIQCIICEDWYHSRHLGVEIPSQNYSEMICEACVGKHQFILHYNGMSLNKVNPSEQVEVEVVKEQSDAKGATEATDNAETSNNVEDPLKLDDDNKMEVASSSDNEQKVEEGNIEQNDNSLNNAESSQVLINNSEVCKMPKTKPTRICTKFWADVEWRNQLCTCPQCLKMYEDQKVMFLLDPQDTVHVYEEKGKAKARELEEREHEDDMKLLNSLDRVPLMEAIAGFNDMKVNLMEYLKKFAENKKVVREEDIREFFSKYDTKKKQKCEIPHYCR

>gi|952513649|gb|KRT80194.1| PHD finger motif containing protein, partial [Oryctes borbonicus]

MESNVEDTSNNDLSADEMETVTLTEVAQSQQSLYADAYAVLGASDDKNCTYSEGYLKRQALYACLTCIPEARTNESKRAGICLACTYHCHDGHELVELYTKRNFRCDCGNSKFPDFTCNLSGNKDETNELNLYNQNFSGVYCTCSRPYPDPDDSVVDEMIQCIICEDWYHTRHLGVDIPSTFAEMICENCVKEHDFLLHYEGLTITRITQDSTDDALDITGDKNATESNEVGSAPNEQSTTSTDQDIIEIDAPNECKKPKNKSGKVCTKFMKDISWRQQLCTCETCTNMYAAQNVTFLTDIHDTVQFYEDKGKATALEDEECAINSVDRVPLMEAIAGYNDLKGHLVEYLKKFQENKKVVREEDIREFFSSMAARKKQKTVIPY

>gi|1233177183|ref|XP_022189957.1| putative E3 ubiquitin-protein ligase UBR7 [Nilaparvata lugens]

MENTPKSESFDSNVENMEDEGNTVTMLDVLEEQQKWETQAYAVLGASDDKNCSYNKGYVKRQALFSCKTCIENEDKPACICLACSYHCHEGHDLIELYTKRNFRCDCGNSLFKGKKCSLEPNKELNELNSYNHNFRGLYCACERPYPDLEESVPDEMLQCIICEDWFHSRHLGGFPTENNFCEVICASCTDRLDFLRYYSGYTMKKITEETNDFSTVNKNVEETNNSSIVDVESPIPNDPAAATVIEEITKPASTSMEKQNVENSKEPEKAEECLLKTMKPVDMPAEEASYWTVGFRKSLCVCQSCQKMYKDLNVSFLTDLSDSVGVYEEEGLKERTKKVSSTNDETTMNALSTLNRVTQIEAIQRYNELQSNLKEYLLKFAENKKVVREEDIHEFFEQMARKKQKVEEVPYFCR

>gi|1574104873|gb|RZF43139.1| hypothetical protein LSTR_LSTR012559 [Laodelphax striatellus]

MENTSKSESFDSNFENNEDEGNTVTMMDVLEEQQKLEEDAYAVLGASDDKNCSYNQGYVKREALFSCKTCIDKDDKPACICLACSYHCHEGHDLIELYTKRNFRCDCGNSLFKGKKCSLEPNKEELNELNNYNQNFRGLYCTCARPYPDLEDSVQDEMLQCTICEDWFHSRHLGSSLPAANNFSEVICGSCTDRLGFLRYYSGQTVKKVVEETNDNSVDVESPVPNVSATVNEETSTPVPMPCTEKQNDENSKEPKKAKECLLKTLKPVEMTGKGATFWMESFRKSLCVCQSCQEMYKDLNVSYLTDVSDMVQAYEERGEKERSNTVGMSNNERTMQALSSLNRVAQIEAIKRYDELQTNLKEYLSKFVENKKVVREEDIHEFFQEMSRKKQKVEEVPYFCH

>gi|1928865726|emb|CAD6207537.1| GSCOCG00010215001-RA-CDS [Cotesia congregata]

MADTSKNVENCEEDNSVTMLDVLQEANQLEEDANAVLGASDDGSCTYNKGYIRQALYACKTCCPNEQDLAGVCLACSFHCHEGHDLVELYTKRNFRCDCGNTKFGEKKCSLDPCKAVENKENKYNQNFKGVYCTCSRPYPDPEDTSDDEMLQCVICEDWYHSKHLGSPEEEIPEDDSFEEVICKDCMSKHDFLRNYAAKYSSSKTKKNSVNNKEDVDVTSPPSETCTMPEKPSTEKTGACFWIEGWRSLLCVCENCKKLYKEQQVSFLLDPTDSVQAYEEAGKVGSSESQYEKGMKALASLGHVQQVNAIEEYNNMKERLKEYLQKFAENKKVVREEDIKEFFSGMASNKKSKVIVPSFCR

>gi|1101357656|ref|XP_018905030.1| PREDICTED: putative E3 ubiquitin-protein ligase UBR7 [Bemisia tabaci]

MADTDEPSVSGKNSNEIVDESNSVTMLEVLAAEKALEEDANAVLGDSDEKNCTYPQGYIKRQALYACLTCTPPDSGRKAGVCLACSYHCHEDHELVELYTKRNFRCDCGTQVFNGKKCSLLADKPLEPNEKNSYNQNFIGLYCSCNRPYPDPEDDTEDIMIQCIICEDWYHFRHLNTEMNSSTDYSEMICETCVRRNPFLLRYVDLHVNKQEINVDVTSVSPIPNPKSVVANVNNPENLPPNEQTVDKETITGAQVETDDSEKQVEVTTEVEGPISSLDKNLTDGTSSSAESAETIPAVVSNCILPVESNELPKIKTLFFNSDWRKQLCKCNNCSMLYKSEGVEFLTDEGDTVLAYEERGKSKEREGQYEQGLRALSSLDRIQQVEALTEYNELKNNLSEYLKKFVENKKIVREEDIKEFFSGLAERKKRKVEPPPYYCR

>gi|1370578478|ref|XP_024215026.1| putative E3 ubiquitin-protein ligase UBR7 isoform X2 [Halyomorpha halys]

MRPPNALQLLQNRLPRVSTVRLDSDMAESSSSEVKDVSDDNTLTLVDFLEEELQLESDANAVLGPSDDKNCTYNRGYLQRQAVYACMTCTPQDSPSSKPAGICLACSYHCHEDHELVELYTKRFFRCDCGNNRFNGKKCNLEPNKTEENEKNVYNQNFRGLYCFCSRPYPDPEDDVSDEMLQCIICEDWYHTRHLKSQVPPETNYAELICGQCMDKLPFLSHYLGLAVFPTSRNSKDNKEDVNIEVDALEGTSSSHVLPDTNSKEELMTIDRNDKTSEEEKTSLLEDKENSQNEAVINVFPTSNESNVVSTVSKDECKLENTRPLSRTEQSAIFWPSNFRSALCCCAKCQDLYAKHNVPWIIDEKDTVQSYEGIGKETQKNSQYEAGLEALARLDRFRQVEAIQNYNDLKSNLSDFLIKFVRQKRVVKKEDIDEFFTDMQARKRQKVGTPPYTCH

>gi|939653928|ref|XP_014275670.1| putative E3 ubiquitin-protein ligase UBR7 isoform X1 [Halyomorpha halys]

MRPPNALQLLQNRLPRVSTVRLDSDMAESSSSEVKDVSDDNTLTLVDFLEEELQLESDANAVLGPSDDKNCTYNRGYLQRQAVYACMTCTPQDSPSSKPAGICLACSYHCHEDHELVELYTKRFFRCDCGNNRFNGKKCNLEPNKTEENEKNVYNQNFRGLYCFCSRPYPDPEDDVSDEMLQCIICEDWYHTRHLKSQVPPETNYAELICGQCMDKLPFLSHYLGLAVFPTSRNSKDNKEDVNIEVDALEGTSSSHVLPDTNSKEELMTIDRNDKTSEEEKTSLLEDKENSQNEKAVINVFPTSNESNVVSTVSKDECKLENTRPLSRTEQSAIFWPSNFRSALCCCAKCQDLYAKHNVPWIIDEKDTVQSYEGIGKETQKNSQYEAGLEALARLDRFRQVEAIQNYNDLKSNLSDFLIKFVRQKRVVKKEDIDEFFTDMQARKRQKVGTPPYTCH

>gi|1475890885|ref|XP_026278475.1| putative E3 ubiquitin-protein ligase UBR7 [Frankliniella occidentalis]

MAEKSSSVEPPVNGQEADDDESNAITMVDVLKEETELEEDANAVLGGSDPLNCTYPQGYVKRQALYACITCIPAGSDQKRGGVCLACSYSCHENHDLVELYTKRNFRCDCGNSQFGSNKCNLEPVKEVNEKNKYNQNFKGVYCTCKRPYPDPEDTSDDEMIQCIICEDWYHGRHLGVNKAIPKDYGEMVCEQCMSKHSFLWNYAGLCLTKATSSDQEVEVDQDPSTFKQTSASDDVEPSTPTIPINGSAGVDFNTPGSSSQKSNIETPIHDGKECILMNFKPVEQLKGATFWPEGWRKQLCLCIKCLENYEANGVSFLTDLQDTVQYYEEQGKAKAANGQASSQYDHAMHALSQLDRTAQIEAIHGYNDMKDQLKEYLQKFAENRKVVREEDIREFFSQMARKRPRVEVSSFCR

>gi|1841800001|ref|XP_034250247.1| putative E3 ubiquitin-protein ligase UBR7 [Thrips palmi]

MAEKSTSEETLRNGADAEDDESNAITMVDVLKEENELEEDANAVLGGSDPQNCTYPQGYVRRQALYACTTCIPANSEQKQGGVCLACSYSCHEGHDLVELYTKRNFRCDCGNSLFGGNKCQLEPVKDINENNKYNQNFNGVYCTCHRPYPDSEDPVDDEMIQCIICEDWYHGRHLGTQKFIPSDYGEMICGSCMSQHSFLWNYAGLCLTKATEGTAETEVNVEMTPQKIAASESSETTSTACAPTTSAGAPNTSSPLKNNGETPSTQSNGVECTLSTFKPVEQHKGATFWPEGWRKQLCTCQKCLEKYEAEAVPYLTDLQDTVQFYEEQGKAKSANGQGNSQYDHAMRALSQLDRTAQIEALHGYNDMKERLKEYLQKFAENKKVVREEDIREFFSQMPRKRAHVELPSNCR

>gi|1834189089|ref|XP_023707660.2| putative E3 ubiquitin-protein ligase UBR7 isoform X1 [Cryptotermes secundus]

MAESSTSTMTSTSKEESVEEDPNVVTMVDVLKEEQELEDDAKAVLGASDDQNCTYSQGYVKRQALYACMTCIPLSDDSLKPGGICLACSYYCHEGHELVELYTKRNFRCDCGNSRFTNNKCNLEPNKSAVNELNSYNQNFRGVYCTCSRPYPDPEDVIDDEMIKCVVCEDWYHGRHLGSNVPTLHDYGEMVCGSCMEKHTFLWQYKREIPDQDGVIKVEVCNGKADAGENVNEIKIEDTRSKIMKTEDRGCLLKELNPVVGHGAVFFSDGWRKYLCRCDSCQTFYETQGLSFLLDDQDTVHAYEEQGKASGETGQPSSRYEQGLKALSSLDRIKQVEAIEEYNNMKIQLKEYLQKFAENKKVVREEDIREFFSGMEARKKQRVDVQMPYFCH

>gi|212508343|gb|EEB12067.1| conserved hypothetical protein [Pediculus humanus corporis]

MTDSSDLNNIEKEDESVVTMVDVLQEENELEEDANAVLGGADDKICTYSKGYIFRQPLYACATCNSSGNGKLGGICLACSYRCHEGHELIELYTKRNFRCDCGNSCFPNTKCNLEIGKDDFNVNNSYNQNFTGIYCNCKRPYPDPDDTIDDEMIQCVICEDWFHKRHLNNNSIPSDYGEMICYECMENHLFLWKYSDLHLKAKSSLDDSSNQKINNSSIKNNIDESQENKNEKESDVNSILNCKIKTVKTENGSGSTFWPDGWRKSLCKCDECLKMYDNEKISFLLSDTDTVQYYENRNKALAKESQYEKGLKALSSLDRVKQVEAIEGYNEMKSELMGYLQKFAESKKVVREEDIREFFSQMQARKKHKVEVPYYCH

>gi|242008008|ref|XP_002424805.1| conserved hypothetical protein [Pediculus humanus corporis]

MTDSSDLNNIEKEDESVVTMVDVLQEENELEEDANAVLGGADDKICTYSKGYIFRQPLYACATCNSSGNGKLGGICLACSYRCHEGHELIELYTKRNFRCDCGNSCFPNTKCNLEIGKDDFNVNNSYNQNFTGIYCNCKRPYPDPDDTIDDEMIQCVICEDWFHKRHLNNNSIPSDYGEMICYECMENHLFLWKYSDLHLKAKSSLDDSSNQKINNSSIKNNIDESQENKNEKESDVNSILNCKIKTVKTENGSGSTFWPDGWRKSLCKCDECLKMYDNEKISFLLSDTDTVQYYENRNKALAKESQYEKGLKALSSLDRVKQVEAIEGYNEMKSELMGYLQKFAESKKVVREEDIREFFSQMQARKKHKVEVPYYCH

>gi|1009540087|ref|XP_015906465.1| putative E3 ubiquitin-protein ligase UBR7 [Parasteatoda tepidariorum]

MAENQNSDVDKETELTMCDVLEEEKELENDANAVLGGSDDKNCSYDQGYVPRQALYACKSCTVDGFAGICLACCYACHDGHEVVELYTKRNFRCDCGNSKFTNSDCVLNPNKQTVNSNNKYNQNFKGLYCICSRPYPDLEDSVPDEMIQCVVCEDWYHGRHLSVEAPDDYFEMICGKCMDSHPFLFSYLSKYDPVIDVVETKNDQQPPTDSIAQKEEAAKDSTSCLLKKLEGGNVKKTGAAFWPENWRKNLCRCSECMNLYRSADCEFLLNLDDTIQAYEEKGKGGENGESQYDKGLSALGQLDRVKQMEAIRAYTSMKGQLKEYLKEFADNKKTVRECDIKEFFERIGKKQKLNNGVPHFCR

>gi|1009540085|ref|XP_015906463.1| putative E3 ubiquitin-protein ligase UBR7 [Parasteatoda tepidariorum]

MAENQNSDVDKETELTMCDVLEEEKELENDANAVLGGSDDKNCSYDQGYVPRQALYACKSCTVDGFAGICLACCYACHDGHEVVELYTKRNFRCDCGNSKFTNSDCVLNPNKQTVNSNNKYNQNFKGLYCICSRPYPDLEDSVPDEMIQCVVCEDWYHGRHLSVEAPDDYFEMICGKCMDSHPFLFSYLSKYDPVIDVVETKNDQQPPTDSIAQKEEAAKDSTSCLLKKLEGGNVKKTGAAFWPENWRKNLCRCSECMNLYRSADCEFLLNLDDTIQAYEEKGKGGENGESQYDKGLSALGQLDRVKQMEAIRAYTSMKGQLKEYLKEFADNKKTVRECDIKEFFERIGKKQKLNNGVPHFCR

>gi|1009560919|ref|XP_015914021.1| putative E3 ubiquitin-protein ligase UBR7 [Parasteatoda tepidariorum]

MAENQNSDVDKETELTMCDVLEEEKELENDANAVLGGSDDKNCSYDQGYVPRQALYACKSCTVDGFAGICLACCYACHDGHEVVELYTKRNFRCDCGNSKFTNSDCVLNPNKQTVNSNNKYNQNFKGLYCICSRPYPDLEDSVPDEMIQCVVCEDWYHGRHLSVEAPDDYFEMICGKCMDSHPFLFSYLSKYDPVIDVVETKNDQQPPTDSIAQKEEAAKDSTSCLLKKLEGGNVKKTGAAFWPENWRKNLCRCSECMNLYRSADCEFLLNLDDTIQAYEEKGKGGENGESQYDKGLSALGQLDRVKQMEAIRAYTSMKGQLKEYLKEFADNKKTVRECDIKEFFERIGKKQKLNNGVPHFCR

>gi|675370429|gb|KFM63331.1| putative E3 ubiquitin-protein ligase UBR7, partial [Stegodyphus mimosarum]

MAESSNSSPKEDTELTMCDVLQEEQELEEDANAVLGGSDDTNCSYDKGYVSRQALYACTTCSVNDQPSGICLACCYACHDGHEVVELYTKRNFRCDCGNSKFPGKTCTLRPEKDPLNLKNKYNQNFRGLYCNCSRPYPDLEDDTPDEMIQCVMCEDWYHGRHLGCEIPDDYFEMICGKCMETHKFLYTYLNNFQNIKVCDDENTIKQENGVQEEKFKTAENKEAVLSIKEENDSQLSTKNQGINNASAVSFQSDSSNVKQEDNICNVKVECDTEKGAENCILKKMETDKIITSGTAFWPENWRTELCHCSSCMDIYLKYDCEYLLNPDDTIQAYEERGKGCATESQYDKGLSALSQMDRVKQMEAIRAYTSMKGQLKEYLKAFADNKRTVSENDIKEFFEGISKRKKLNNGVPYFCR

>gi|1866157490|ref|XP_035218623.1| putative E3 ubiquitin-protein ligase UBR7 [Stegodyphus dumicola]

MAESSNSSPKEDTELTMCDVLQEEQELEEDANAVLGGSDDTNCSYDKGYVSRQALYACTTCSVNDEPGGICLACCYACHDGHEVVELYTKRNFRCDCGNSKFPGKTCTLRPEKDPLNLKNKYNQNFRGLYCNCSRPYPDLEDDTADEMIQCVMCEDWYHGRHLGCEIPDDYFEMICGKCMETHKFLYTYLNNFQNIKVCDDENTIKQENGVQEEKFKTEESIESVISVKEENDSQLNTESQEISSASEVNFQSDSSVVKQEANVCNVKKESATDEVKQVVENCILKKMETDKIITSGTAFWPENWRKELCHCSSCMDMYLKYDCEFLLNPDDTIQAYEERGKGCATESQYDKGLSALSQMDRVKQMEAIRAYTSMKGQLKEYLKAFADNKRTVSENDIKEFFEGISKRKKLNNGVPYFCR

>gi|1557996377|gb|RWS16479.1| putative E3 ubiquitin-protein ligase UBR7-like protein [Dinothrombium tinctorium]

MAENSKSKAETSSALNASERGEMPLEDEENDNVVTMVDVLNEEQELEDSAFAVLAGSDDKNCTYNLGYLNRQALYACKTCKTKDSRPAGICLACSLECHEGHEVYELYTKRNFRCDCGNDLFPDNKCKLCANKKPTNDDNKYDHNFFGRYCICDRPYPDVENDDDDQMIQCVVCEDWFHGRHLGGALPENDEFAELICDSCMKRLSFLWFYYTASNSSPNQEEKEIEVTRPATSALCDSDSGFESSCDSVSATSCKLEKLRKNKNVSQMTGPTFWDEDWRSELCKCIHCLAMYDQQCCSFLTNEEDTVHYYESQGKERNIKISQYERGLKEINKIDRVKSIEAIEECNAMTNELKDYLKKFAENKKVVRQEDIMEFFEGLKARKRQKVSIPYTCR

>gi|1005953965|ref|XP_015785376.1| putative E3 ubiquitin-protein ligase UBR7 [Tetranychus urticae]

MSESEVKSLEPVSSSLDATGVVKKEEAIDDLQDPEAAGDEDCENVITMMDVLEEEKQLEDDAFAVLGGSDEKDCTYLRGYVVRQALYACNTCKPSAGEPNGICLACTYACHEGHDLYELYTKRSFRCDCGNSKMAGSKCNLCPTKSATNPDNKYNHNFSGLYCTCNRPYPDPDNDDAMYQCIVCEDWFHDKHLESEIPEEKGFAEMVCSPCMTKLDFLWYYYKDTLKSSEDNAQINQKQQASSQADQKPIVSSAQEPSSSENNIAPNPSTSSATSSQTEETESDSKSSNADNIDSGIESSCDSIVSPSSTNCKLESLQSSITIDTLKGATFWEEGWRSNLCKCTACLKKYSSLGCAFICDETDTVSFYEKQGKEKSIQISQYERGLNEINKMDRVSTIEAIQECNFMAEELKTYLKKFADNKKVVRREDIDDFFEGLKARKRSRYDVGY

>gi|1707851423|ref|XP_029844039.1| putative E3 ubiquitin-protein ligase UBR7 [Ixodes scapularis]

MADGEGSGTQLTSPEIQDENSLTLQDILDQEQELQDDANAVLGGSDDKNCTYEKGYVRRQALYACSTCVSPDSRPAGICLACSYACHEGHQLYELYTKRNFRCDCGNSRFPSTNPCRLCPAKDATNAGNDYNQNFHGRYCTCGRPYPDPEDDVDDEMLQCVICEDWYHGRHLGGDVPGNRDYSEVICLGCMSRHPFLCHYLAHAVALCKAENDETFSVDDGACNGSSANGLSGMTPVDLKPSVGTKGIGNQDGNVMPCSKGFLGAAATNGTCGGCNTGASGNVPLTSGMPKVETHQSSSGCVLRRLSGAGAREGPTGQRCAFWPPGWRSQLCRCHDCMAMYEAQHCQFLLDEDDTVQSYEERGKAAQPGPETGDPLMSALGTLGRVQQIEAIHGYNNLKTELTDFLKKFATSKTVVREEDVREFFDQMQARKRQKVSAVPYFCR

>gi|215494701|gb|EEC04342.1| conserved hypothetical protein [Ixodes scapularis]

MADGEGSGTQLTSPEIQDENSLTLQDILDQEQELQDDANAVLGGSDDKNCTYEQGYVRRQALYACSTCVSPDSRPAGICLACSYACHEGHQLYELYTKRNFRCDCGNSRFPSTNPCRLCPAKDATNAGNNYNQNFHGRYCTCGRPYPDPEDDVDDEMLQCVMCEDWYHGRHLGGEVPGNRDYSEVICLGCMSRHPFLCHYLAHGERGVKKTVALCKAENDETFSVDDGACNGSSANGLSGMTPVDLKPSVGTKGGDDFFPLPSGISSSKRFPPKQPEKPNVETHQSSSGCVLRRLSGAGAREGPTGQRCAFWPPGWRSQLCRCHDCMAMYEAQHCQFLLDEDDTVQSYEERGKAAQPGPETGDPLMSALGTLGRVQQIEAIHGYNNLKTELTDFLKKFATSKTVVREEDVREFFDQMQARKRQKVSAVPYFCR

>gi|1930402536|ref|XP_037271851.1| putative E3 ubiquitin-protein ligase UBR7 [Rhipicephalus microplus]

MADDGKTSESAPSTSIDLQDENSVTLQDVLDEEQELEDDADAVLGGSDDKNCTYDQGYVKRQALYACNTCTGPDSQPAGVCLACSYACHEGHNLYELYTKRNFRCDCGNASFPESNPCRLCPRKAVRNYENKYNHNFHGMYCTCKRPYPDPDDDVEDEMLQCIMCEDWYHGRHIGGAMPANRDYYEVVCTGCMSKHPFLWQYFAHEMALLEEKDVNICEEDTEKQHADGSPAASTTTVENGVKVEGGNTENGVASARENGTDAVAIKEEGHPNQQTAGSSGSCSEVKVEKMAGDGSQLSSASECILKKLRSVGDDSKKPHDACAYWPRGWRSRLCHCIQCLAMYNEQKCLFLLDEEDTVQSYEEKGKAARAQVPEGDPLMSALGSLGRVQQIEAIHGYNNLKTQLVDFLKKFADNRKVVREEDVREFFSGMEARKRRKTSAVPYFCR

>gi|1933841429|ref|XP_037580715.1| putative E3 ubiquitin-protein ligase UBR7 [Dermacentor silvarum]

MADDGKFSESAPSTSIDLQDENSVTLQDVLDEEQELEDDADAVLGGSDDKNCTYDQGYIKRQALYACGTCTGPDSQPAGVCLACSYACHEGHNLYELYTKRNFRCDCGNASFPANNPCRLCPRKAVRNDENKYNHNFHGVYCTCKRPYPDPDDDVEDEMLQCIMCEDWYHGRHIGGDMPPNRDYYEVVCNGCMSKHPFLRWYFRAWHAWLPFAEMSLLEKDASLCEDATEKKQAEESSSALATTTENGIKAEGSVENGVGTSRENGTVATDSTAKEEHPSQPTAGSSGSRDEVKVEKLAGESGLLSSVSQCILQRLQSNTDDVKEEPQNECAYWPRGWRSRLCHCIKCLAMYNEQHCLFLLDEEDTVQSYEEKGKAARAQAPIGDPLMSALGTLGRVQQIEAIHGFNNLKTELIDFLKKFADNRKVVREEDVREFFSGMEARKRQKTSAVQYFCR

>gi|1941214867|ref|XP_037902959.1| putative E3 ubiquitin-protein ligase UBR7 [Hermetia illucens]

MSDEVKIEDVPPTDSESNVMDDSSVTMMDVLKQQEKLEEESAAVLGGSDEKSCTYERGYIKRQALYSCLTCCPEACNDPSKRAGVCLACSYQCHENHELVELYTKRNFRCDCPTSRIPGQKCTFAETSSPNEPKHEPNKDNSYNQNFFGLYCTCKRPYPDPEDSIPDEMIQCVLCEDWYHTRHLNAPIPAADSYSEMICDVCMEKHSFLKDYSGLAVNKVEPTNESANVSVLNCTTDEEKLRADLDKSIADIMPNISSSDDTMEDSATISGTHDEGVGEPATKKPKLDEDAGKCRRPPASLGYKGGAVFWPDNWRSSLCSCQTCSQIYESQKVSFLLDPEDTVQFYEENGLKKVVETDYERGIRAFSTMGRTQQIDALTEYNRMKEKLQEFLRTFAVNKKVVTEEDINTFFQGMKNEKNANVGQPYFCR

>gi|157019184|gb|EAA05935.4| AGAP009512-PA, partial [Anopheles gambiae str. PEST]

ANYKMEQKAEQDQDTSYVTMPDILQEQEELEETSRAVLGGSDEKNCTYTKGYVGRQALYACLTCVPEARGIESKRSGICLACSLQCHDNHELLELYTKRHFRCDCGGPRMPEVKCKLEPRKEEANDRNRYNQNFSGLYCVCHRPYPDPDDDVQDEMVQCVVCEDWYHMRHLDLDEPKSSKDYGEMVCGGCMEANPFLQNYVGKIEDPNKTTLDDTVQVDVTGLDESNDTAGPSEPKQRRLDDSKAPAGEEPQKQKEEPVVSLDICTKPVVVADGEKVYKKGAAFWVEGWRKYLCQCKACVELYKKHGVEYLLDEQDTVKYYEDVGKQKHGSNGSAYEQGMQMLGQLDRVTQVDMLTEYNRMTSRLREFLDQFVTNQQVVTRDDINEFFAKLNKERRESGSSGPPPYFCR

>gi|158288301|ref|XP_310183.4| AGAP009512-PA, partial [Anopheles gambiae str. PEST]

ANYKMEQKAEQDQDTSYVTMPDILQEQEELEETSRAVLGGSDEKNCTYTKGYVGRQALYACLTCVPEARGIESKRSGICLACSLQCHDNHELLELYTKRHFRCDCGGPRMPEVKCKLEPRKEEANDRNRYNQNFSGLYCVCHRPYPDPDDDVQDEMVQCVVCEDWYHMRHLDLDEPKSSKDYGEMVCGGCMEANPFLQNYVGKIEDPNKTTLDDTVQVDVTGLDESNDTAGPSEPKQRRLDDSKAPAGEEPQKQKEEPVVSLDICTKPVVVADGEKVYKKGAAFWVEGWRKYLCQCKACVELYKKHGVEYLLDEQDTVKYYEDVGKQKHGSNGSAYEQGMQMLGQLDRVTQVDMLTEYNRMTSRLREFLDQFVTNQQVVTRDDINEFFAKLNKERRESGSSGPPPYFCR

>gi|167872358|gb|EDS35741.1| mlo2 [Culex quinquefasciatus]

MDTKENATASSGSSKLDESSVTMLDVLNEQNELEAESDAVLGGSDEKNCTYALGYIGRQALYACVTCSPESAVGEEKRAGVCLACSYHCHEGHELVELYTKRNFRCDCGGKRMPDVRCKLDPIKLDENEGNQYNQNFGGLYCTCHRPYPDPEDTVPDEMIQCVVCEDWYHTRHLENDEPKNSKDYAEMVCHLCMERVVPLRNYVGKLEDCNRTLLNETVQLDVTGLDDSVATAADTSVAPSESEEDLNESKRIKLDICSKPAPEEGDKAYKKGATFWHDGWRKQLCRCTECMECYRKLKVEFLLDEKDTVQWYEENGRAKREENGSTYEQGMQMWNKMGRVQQVEILSGYNLMKDRLASFLDTFVTNQQVVTEKDIKEFFEKLNKERRETNMAPPSSCR

>gi|1949586050|ref|XP_038107087.1| putative E3 ubiquitin-protein ligase UBR7 [Culex quinquefasciatus]

MDSKENATASSGSSKLDESSVTMLDVLNEQNELEAESDAVLGGSDEKNCTYALGYIGRQALYACVTCSPESAVGEEKRAGVCLACSYHCHEGHELVELYTKRNFRCDCGGKRMPDVRCKLDPIKLDENEGNQYNQNFGGLYCTCHRPYPDPEDTVPDEMIQCVVCEDWYHTRHLENDEPKNSKDYAEMVCHLCMERVVPLRNYVGKLEDCNRTLLNETVQLDVTGLDDSVATAADTSVAPSESEEDLNESKRIKLDICSKPALEEGDKAYKKGATFWHDGWRKQLCRCTECMECYRKLKVEFLLDEKDTVQWYEENGRAKREENGSTYEQGMQMWNKMGRVQQVEILSGYNLMKDRLASFLDTFVTNQQVVTEKDIKEFFEKLNKERRETNMAPPSSCR

# 4. Antibody preparation

Anti-UBR7 was prepared by predicting the antigenic determinant through BepiPred-2.0, synthesizing the peptide (Peptide 1: CKRPYPDPEDTSDDE; Peptide 2: Cys+NTPGSSSQKSNIETP)

### 4.1 Project Information

Antigen Name: UBR7

Peptide Sequence #1: CKRPYPDPEDTSDDE

Purity: 93.69%

Peptide Sequence #2: Cys+NTPGSSSQKSNIETP

Purity: 97.24%

Immunogen: Peptide-KLH conjugate

Host Strain: New Zealand Rabbit

Purification Step: Affinity purification against the antigen

### 4.2 Product Information

## Supplementary table 4: Information about antibody preparation products

**Table S4. Information about antibody preparation products**

| **Product** | **Animal** | **Volume** | **Quantity** | **Concentration** | **Form** |
| --- | --- | --- | --- | --- | --- |
| Pre-immune | serum | 1# | 0.5ml | / | Liquid |
| Pre-immune | serum | 2# | 0.5ml | / | Liquid |
| Antiserum |  |  | 5ml | / | Liquid |
| Purified Antibodies CKRPYPDPEDTSDDE |  | 3ml | 2mg | 0.67mg/ml | Liquid |
| Purified Antibodies Cys+NTPGSSSQKSNIETP |  | 3ml | 2.04mg | 0.68mg/ml | Liquid |
| Peptide CKRPYPDPEDTSDDE |  |  | 2mg | / | Lyophilized |
| Peptide Cys+NTPGSSSQKSNIETP |  |  | 2mg | / | Lyophilized |

Buffer: Phosphate Buffered Saline (PBS, pH 7.4)

### 4.3 QC (Quality Control) Results

Indirect ELISA:

Coating Antigen: Peptide-BSA conjugate

Coating Concentration:5 μg/ml, 100μl/well

Coating Buffer: CBS, pH9.6

Secondary Antibody: Peroxidase AffiniPure Goat Anti-Rabbit IgG (H+L)

Final ELISA results against peptide #1：CKRPYPDPEDTSDDE

Final ELISA results against peptide #2：Cys+NTPGSSSQKSNIETP

## Supplementary table 5: Quality Control of antibody preparation products

**Table S5. Quality Control of antibody preparation products**

| **Sample-1** | **Dilution** | **OD450** |  | **Sample-2** | **Dilution** | **OD450** |
| --- | --- | --- | --- | --- | --- | --- |
| Rabbit #1 Antibody | 2000 | 3.94 |  | Rabbit #2 Antibody | 2000 | 3.73 |
|  | 4000 | 3.18 |  |  | 4000 | 3.64 |
|  | 8000 | 1.91 |  |  | 8000 | 3.47 |
|  | 16000 | 1.08 |  |  | 16000 | 2.7 |
|  | 32000 | 0.75 |  |  | 32000 | 1.93 |
|  | 64000 | 0.42 |  |  | 64000 | 1.27 |
|  | 128000 | 0.31 |  |  | 128000 | 0.92 |
| Negative | 1000 | 0.04 |  | Negative | 1000 | 0.05 |
| Blank | N/A | 0.03 |  | Blank | N/A | 0.04 |

The titer is the highest dilution with S/N(Signal/ Negative) >=2,

Negative control is Pre-immune serum, Blank is PBS

# 5. RT-qPCR standard curve preparation

### 5.1. Standard preparation

The primers used to prepare the standard were all primers for RT-qPCR (See Table 1 for details.).

q-UBR7-F: CAGATGATGACGAAAGTAACGC

q-UBR7-R: AGCAAGGCATACACCACCTC

q-Actin-F: GGTATCGTCCTGGACTCTGGTG

q-Actin-R: GGGAAGGGCGTAACCTTCA

q-TSWV-F: CTTGCCATAATGCTGGGAGGTAG

q-TSWV-R: TCCCGAGGTCTTTGTATTTTGC

According to the instructions of a 2 × TransTaq® High Fidelity PCR SuperMix I (-dye) kit (TransGen, Beijing, China), PCR amplification was conducted in an Applied Biosystems Veriti™ Dx 96-Well Fast Thermal Cycler (Thermo, Massachusetts, USA). The PCR amplification products were electrophoresed at a constant pressure of 120 V until the bands reached 2/3 of the agarose gel, the specificity of the amplified primer products was checked by observation on a gel transilluminator and the target fragment was cut off from the agar block. The agar blocks containing the target fragments were purified and recovered in strict accordance with the instructions in the EasyPure® Quick Gel Extraction Kit (Transgen, Beijing, China). The recovered product was used as a standard. The DNA standards were sequentially diluted to 5 orders of magnitude for the plotting of standard curves for RT-qPCR.

### 5.2. Standard curve drawing

The standard curve was depicted with the absorbance as the x-axis and the concentration as the y-axis.

## Supplementary table 6: Data related to standard curve

**Table S6. Data related to standard curve**

| **Target gene** | **Well** | **Well Type** | **Threshold (dR)** | **Ct (dR)** | **Rel. Quant. to Std.  (for validation)** | **RSq (dR)** | **Slope (dR)** | **Regression equation** | **Correlation coefficient  (R^2^)** | **PCR efficiency  (%)** |
| --- | --- | --- | --- | --- | --- | --- | --- | --- | --- | --- |
| *UBR7* | B3 | Standard | 472.624 | 8.90 | 1.00E-03 | 0.998 | -3.201 | y = -3.201Log(x) ± 0.58 | 0.998 | 105.3 |
|  | B4 | Standard | 472.624 | 12.18 | 1.00E-04 | 0.998 | -3.201 |  |  |  |
|  | B5 | Standard | 472.624 | 15.64 | 1.00E-05 | 0.998 | -3.201 |  |  |  |
|  | B6 | Standard | 472.624 | 18.85 | 1.00E-06 | 0.998 | -3.201 |  |  |  |
|  | B7 | Standard | 472.624 | 21.58 | 1.00E-07 | 0.998 | -3.201 |  |  |  |
| *Actin* | B3 | Standard | 467.157 | 8.90 | 1.00E-03 | 1.000 | -3.256 | y = -3.256Log(x) ± 0.78 | 1.000 | 102.8 |
|  | B4 | Standard | 467.157 | 12.29 | 1.00E-04 | 1.000 | -3.256 |  |  |  |
|  | B5 | Standard | 467.157 | 15.62 | 1.00E-05 | 1.000 | -3.256 |  |  |  |
|  | B6 | Standard | 467.157 | 18.69 | 1.00E-06 | 1.000 | -3.256 |  |  |  |
|  | B7 | Standard | 467.157 | 21.99 | 1.00E-07 | 1.000 | -3.256 |  |  |  |
| *TSWV N* | D1 | Standard | 454.454 | 8.82 | 1.00E-01 | 0.990 | -3.302 | y = -3.302Log(x) ± 4.88 | 0.990 | 100.8 |
|  | D2 | Standard | 454.454 | 10.67 | 1.00E-02 | 0.990 | -3.302 |  |  |  |
|  | D3 | Standard | 454.454 | 14.65 | 1.00E-03 | 0.990 | -3.302 |  |  |  |
|  | D4 | Standard | 454.454 | 18.29 | 1.00E-04 | 0.990 | -3.302 |  |  |  |
|  | D5 | Standard | 454.454 | 21.52 | 1.00E-05 | 0.990 | -3.302 |  |  |  |
|  |  |  |  |  |  |  |  |  |  |  |
| Note： y is defined as concentration and x is the Ct values. | | | | | | | | | | |

# 6. Surface plasmon resonance related data

### 6.1. Prokaryotic expression

**6.1.1 Target sequence information**

Name: UBR7-domino

Expression systems: E.coli Expression System

Gene Sequence:

CTAAATTGCACTTACCCTCAGGGCTATGTGAAGCGACAAGCTCTTTATGCTTGTATTACATGCATTCCAGCTGGCTCTGACCAAAATAGAGGTGGTGTATGCCTTGCTTGCAGTTACAGCTGCCATGAGAATCACGATCTTGTAGAGCTGTACACCAAGAGGAATTTTCGCTGTGATTGTGGCAACTCACAATTTGGCAGCAACAAGTGCAACTTGGAGCCAGTGAAAGAAGTAAATGAAAAGAACAAGTACAATCAAAATTTTAAAGGAGTGTACTGCACTTGCAAGAGACCATATCCTGATCCAGAAGACACCAATGATGATGAAATGATCCAATGTATAATTTGTGAAGACTGGTATCATGGCAGGCACTTGGGAGTTAACAAAGCAATTCCAAAAGATTACGGCGAAATGGTCTGTGAACAGTGCAT

Amino acid sequence:

LNCTYPQGYVKRQALYACITCIPAGSDQNRGGVCLACSYSCHENHDLVELYTKRNFRCDCGNSQFGSNKCNLEPVKEVNEKNKYNQNFKGVYCTCKRPYPDPEDTNDDEMIQCIICEDWYHGRHLGVNKAIPKDYGEMVCEQC*

**6.1.2 Codon-optimized DNA sequence**

***>*** ***Gene coding for the target protein*（UBR7-domino）**

AtgggcagcagccatcatcatcatcatcacagcagcggcctggtgccgcgcggcagccatatgCTGAATTGTACATATCCGCAGGGATATGTGAAGAGACAGGCACTGTATGCATGTATTACCTGCATTCCGGCAGGAAGCGATCAGAATCGGGGCGGTGTTTGTCTGGCATGTAGTTATAGCTGTCATGAAAATCATGACTTAGTTGAACTGTATACAAAGCGTAATTTCCGTTGTGATTGTGGTAACAGTCAGTTTGGGAGCAATAAATGTAATCTGGAGCCTGTTAAGGAGGTGAACGAAAAAAATAAATATAATCAGAATTTTAAAGGTGTTTATTGTACCTGTAAACGTCCGTATCCGGATCCGGAAGATACCAATGATGATGAAATGATTCAGTGTATTATTTGTGAAGATTGGTATCATGGTCGTCATCTGGGTGTTAATAAAGCAATTCCGAAAGATTATGGTGAAATGGTTTGTGAACAGTGTTAAGAATTC

**6.1.3 Expression vector**

The cDNA sequence was cloned in PET28b expression vector.

Cloning strategy: NdeI/EcoRI

**>** **UBR7-domino (164AAs; 18.67KDa; pI:6.57)**

MGSSHHHHHHSSGLVPRGSHMLNCTYPQGYVKRQALYACITCIPAGSDQNRGGVCLACSYSCHENHDLVELYTKRNFRCDCGNSQFGSNKCNLEPVKEVNEKNKYNQNFKGVYCTCKRPYPDPEDTNDDEMIQCIICEDWYHGRHLGVNKAIPKDYGEMVCEQC*

Features: GS linker with 6*His tag：[1-13]；

Thrombin site [14-19]；

## Supplementary figure 1. Protocol for prokaryotic expression


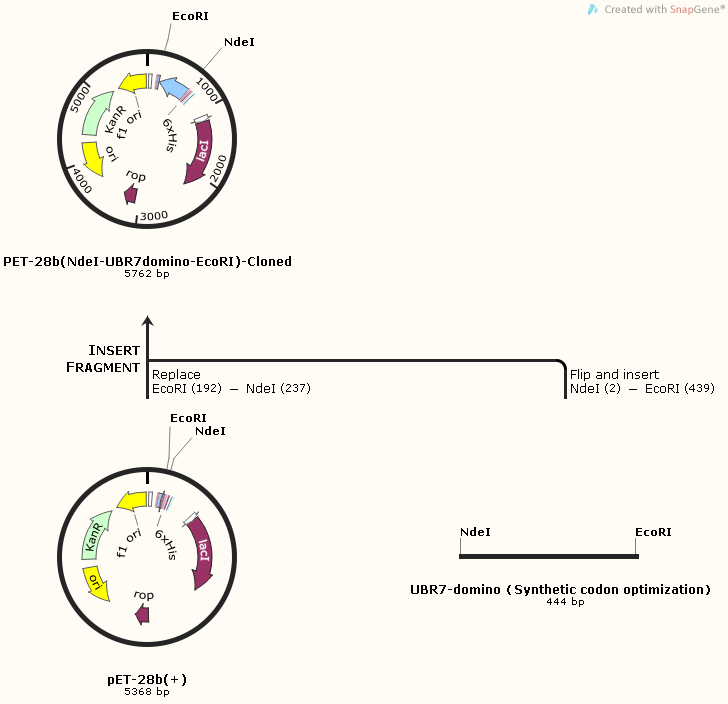


**Figure S1. Protocol for prokaryotic expression**

**6.1.4 Results of the expression tests**

## Supplementary figure 2. Expression tests of the target protein UBR7-domino


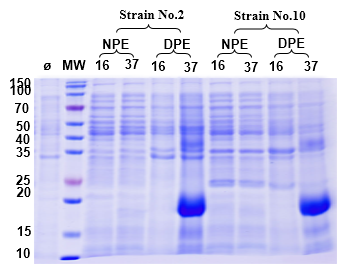


**Figure S2. Expression tests of the target protein UBR7-domino**

**MW.** Molecular weight marker. **Ø.** Non-induced bacteria culture (negative control).

**16 and 37.** Incubation temperature (°C) during induction with IPTG.

Induction with IPTG 1mM during 16h at 16°C, or during 4h at 37℃.

**No.2: T7E strain No.10: Rosetta strain**

UBR7-domino had expressed. Optimal expression condition was described as below.

| **Name** | **Strain No.** | **Temperature** | **Induction Time** |
| --- | --- | --- | --- |
| UBR7-domino | No.10 | 37°C | 4h |

### 6.2 L scale-up purification

**6.2.1 Parameters of the purification tests**

For DPE purification,

-Lysis buffer: PBS, pH7.5, 10% Glycerol

-Washing buffer: PBS, pH7.5, 1% Triton X 100, 5 mM EDTA

-Denaturing buffer: PBS, pH7.5,4 Murea

-Dialysis buffer: PBS, pH7.5

-Final sample QC: qualitative by SDS-PAGE, quantitative by Bradford method.

**6.2.2 Results of the purification tests**

## Supplementary figure 3. Reducing-PAGE analysis. Final sample QC. 2µg of sample loaded


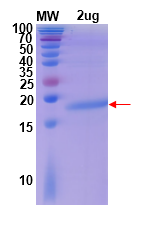


**Figure S3. Reducing-PAGE analysis.**

Final sample QC. 2µg of sample loaded

### 6.3. Surface plasmon resonance

**6.3.1 Parameters**

## Supplementary table 7: Experimental parameters of SPR

**Table S7. Experimental parameters of SPR**

| **Project details** | |
| --- | --- |
| Ligand | LOC113206563 |
| Analytes | Protein extracted from TSWV-infected *Nicotiana* *benthamiana* L. |
| Running buffer | HEPES |
| Regeneration solution | 10mM Glycine-HCl/ 0.25%SDS |
| Association and dissociation flow rate | 20μL/min, Association 240 s, Dissociation 300 s |
| Temperatures | 25 ℃ |
| Sensor Chip | Sensor Chip COOH (Nicoya) |
| Open SPR | Open SPRTM (Nicoya) |

**5.3.2 Sample loading result**

Sample on sample signal diagram

## Supplementary figure 4. Sample signal diagram


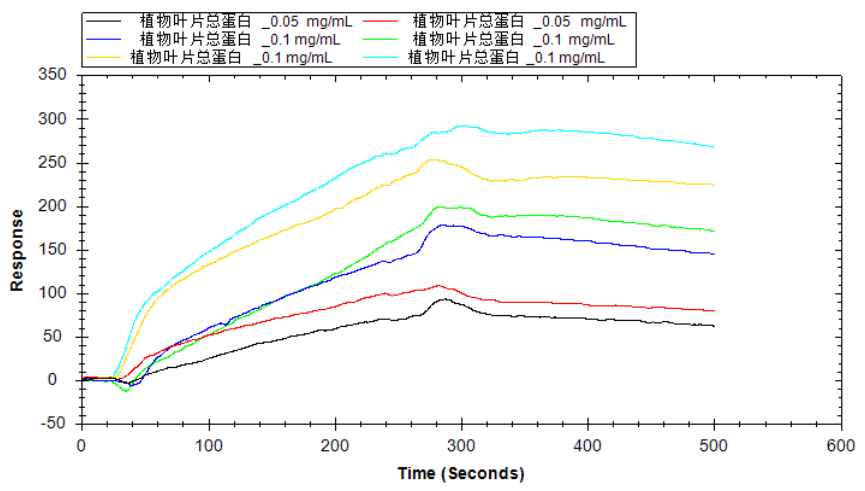


**Figure S4. Sample signal diagram**

植物叶片总蛋白：Total proteins were extracted from leaves

## Supplementary figure 5. Sample elution

**
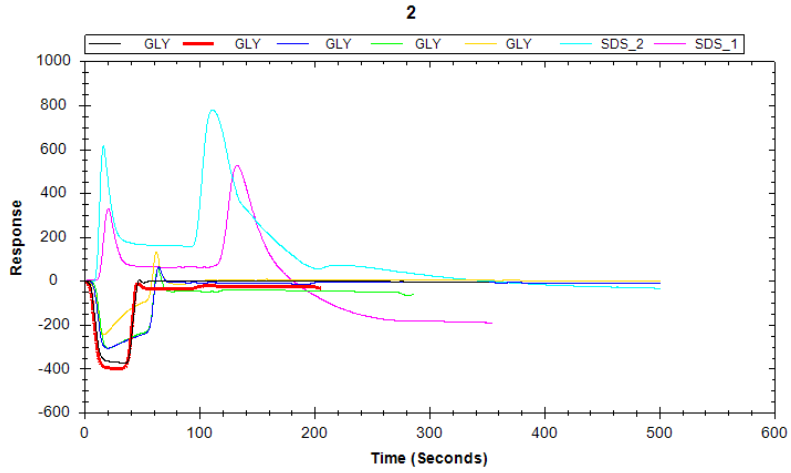
**

**Figure S5. Sample elution**

### 6.4 Protein mass spectrometry

## Supplementary table 8: Peptides interacting with UBR7-Domino identified by SPR and LC-MS/MS

| **Table S8. Peptides interacting with UBR7-Domino identified by SPR and LC-MS/MS** | | | | | | | | | | | | |
| --- | --- | --- | --- | --- | --- | --- | --- | --- | --- | --- | --- | --- |
| **Accession** | **Number** | **Peptide** | **Unique** | **Matches** | **Modification** | **Observed** | **Mr(expt)** | **Mr(calc)** | **ppm** | **Miss** | **Score** | **Query** |
| gi|284810746 | 1 | K.VCTVLK.S |  | 1 |  | 360.2108 | 718.4071 | 718.4048 | 3.25 | 0 | 14 | 33 |
|  | 2 | R.RLDSLIR.V | U | 3 |  | 436.7717 | 871.5288 | 871.5239 | 5.65 | 1 | 36 | 950 |
|  | 3 | R.IGATD->M<-TFR.R | U | 4 | Oxidation (M) | 514.2495 | 1026.4843 | 1026.4804 | 3.81 | 0 | 30 | 2285 |
|  | 4 | K.FYE->M<-FGVKK.Q | U | 1 | Oxidation (M) | 582.7925 | 1163.5705 | 1163.5685 | 1.73 | 1 | 23 | 3753 |
|  | 5 | R.IGATD->M<-TFRR.L | U | 1 | Oxidation (M) | 395.2029 | 1182.5868 | 1182.5815 | 4.46 | 1 | 4 | 4020 |
|  | 6 | K.ENIVALLTQGK.D | U | 1 |  | 593.3485 | 1184.6825 | 1184.6765 | 5.06 | 0 | 38 | 4035 |
|  | 7 | K.AFE->M<-NEDQVK.K | U | 2 | Oxidation (M) | 613.7744 | 1225.5343 | 1225.5285 | 4.72 | 0 | 40 | 4467 |
|  | 8 | K.AFE->M<-NEDQVKK.G | U | 1 | Oxidation (M) | 452.2161 | 1353.6266 | 1353.6234 | 2.30 | 1 | (21) | 6246 |
|  | 9 | K.AFE->M<-NEDQVKK.G | U | 1 | Oxidation (M) | 677.8215 | 1353.6285 | 1353.6234 | 3.74 | 1 | 23 | 6247 |
|  | 10 | K.EYAAILSSSNPNAK.G | U | 4 |  | 732.8737 | 1463.7329 | 1463.7256 | 5.00 | 0 | 61 | 8132 |
|  | 11 | K.TFCLENLDQIKK.M | U | 1 |  | 754.8953 | 1507.7760 | 1507.7704 | 3.66 | 1 | 11 | 8860 |
|  | 12 | K.LTKENIVALLTQGK.D | U | 1 |  | 764.4614 | 1526.9083 | 1526.9032 | 3.37 | 1 | 58 | 9133 |
|  | 13 | K.GSIA->M<-EHYSETLNK.F | U | 1 | Oxidation (M) | 798.3757 | 1594.7369 | 1594.7297 | 4.53 | 0 | 25 | 10133 |
|  | 14 | K.GSIA->M<-EHYSETLNK.F | U | 1 | Oxidation (M) | 532.5870 | 1594.7391 | 1594.7297 | 5.91 | 0 | (14) | 10134 |
|  | 15 | K.GKEYAAILSSSNPNAK.G | U | 1 |  | 550.6226 | 1648.8459 | 1648.8420 | 2.34 | 1 | 1 | 10764 |
|  | 16 | R.LVEETGNSENLNTIK.S | U | 4 |  | 830.9269 | 1659.8393 | 1659.8315 | 4.71 | 0 | 80 | 10898 |
|  | 17 | R.VRLVEETGNSENLNTIK.S | U | 1 |  | 639.3438 | 1915.0094 | 1915.0010 | 4.38 | 1 | 109 | 13492 |
|  | 18 | K.IASHPLIQAYGLPLDDAK.S | U | 2 |  | 961.5263 | 1921.0379 | 1921.0309 | 3.68 | 0 | 84 | 13533 |
|  | 19 | K.IASHPLIQAYGLPLDDAK.S | U | 3 |  | 641.3545 | 1921.0416 | 1921.0309 | 5.60 | 0 | (57) | 13534 |

## Supplementary table 9: The information of protein mass spectrometry

After surface plasmon resonance, the peptide sequence was identified by LC-MS/MS, QE by BGI Genomics. (See Additional file 2: Supplementary table 9.)

**5.4.1 Overview of Results**

A total of 31 proteins were identified in this project, among which 8 proteins contained modification sites.

**5.4.2 Search parameters**

## Supplementary table 10. Search parameters of SPR

**Table S10. Search parameters of SPR**

| **Parameter** | **Parameter Details** |
| --- | --- |
| Type of search | MS/MS Ion Search |
| Enzyme | Trypsin |
| Fixed modifications | Carbamidomethyl (C) |
| Variable modifications | Gln-pyro-Glu (N-termQ) , Oxidation (M) |
| Mass values | Monoisotopic |
| Protein mass | Unrestricted |
| Peptide mass tolerance | -/+ 15 ppm |
| Fragment mass tolerance | -/+ 20mmu |
| Maxmissed cleavages | 2 |
| Instrument type | Default |
| Number of queries | 19,758 |
| Database | ncbi_ *Nicotiana benthamiana* (3,172 sequences; 1,442,534 residues) |

**5.4.3 Score distribution**


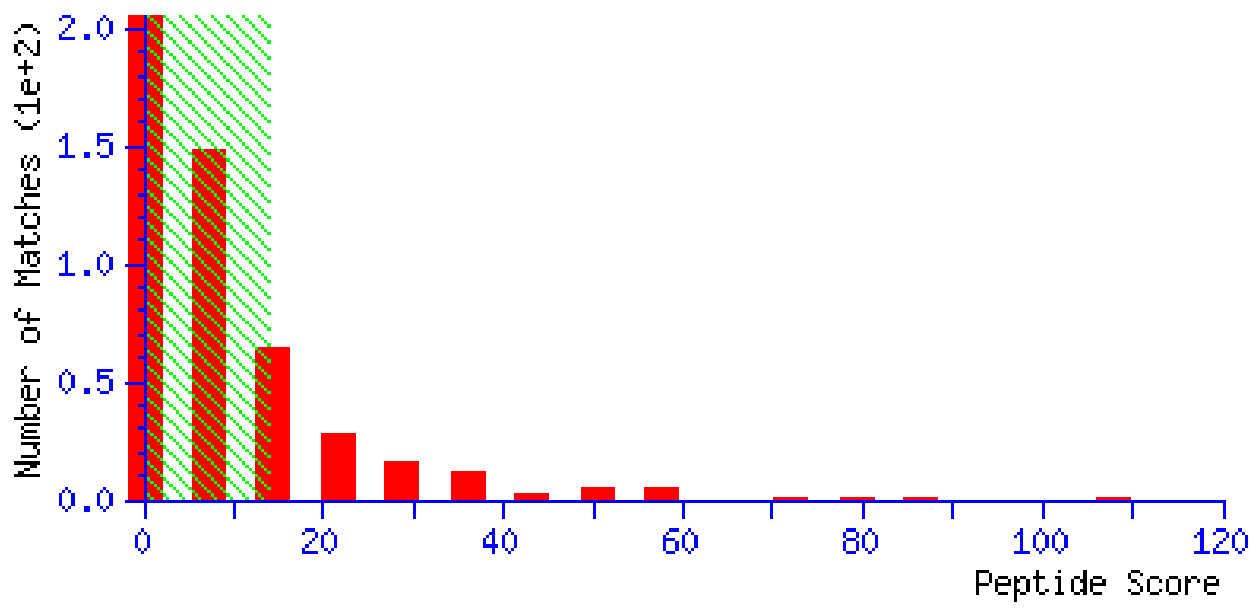


Peptide score distribution. Ions score is −10 log(P), where P is the probability that the observed match is a randomevent. Individual ions scores > **14** indicate **identity** or **extensive homology** (*p*<0.05).


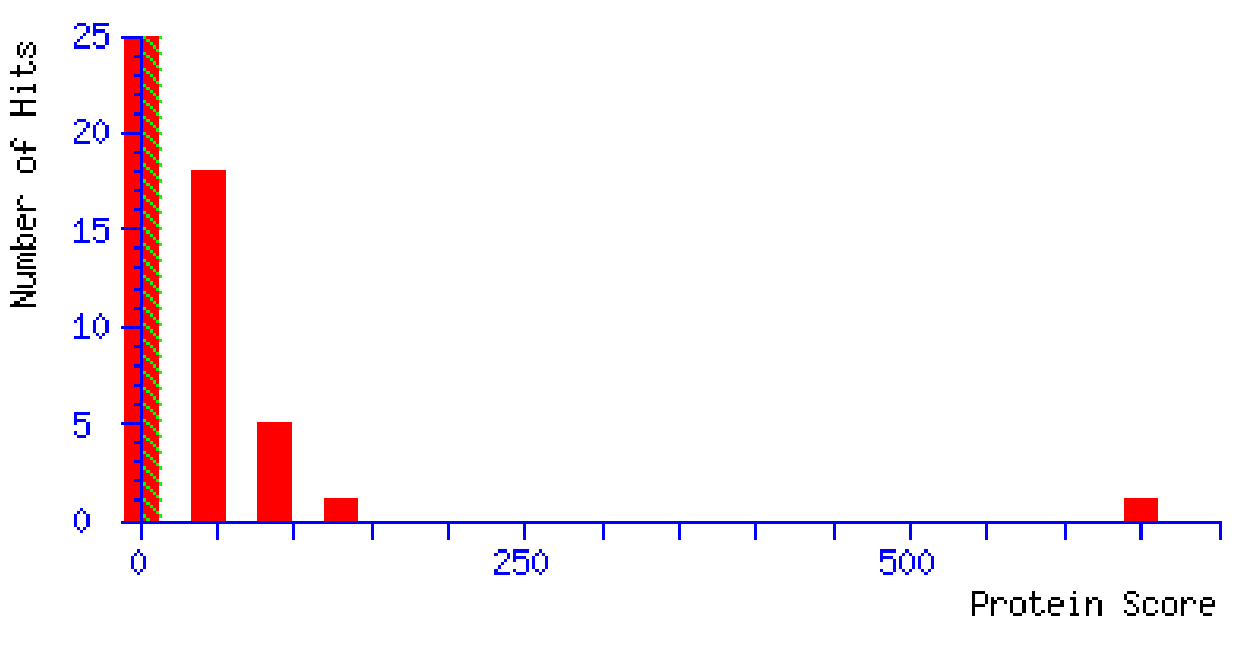


[**Deprecated**] Score distribution for family members in the first 50 proteins. Protein scores are derived from ions scores as a non-probabilistic basis for ranking protein families.

**5.4.4 Decoy search summary**

## Supplementary table 11: Decoy search summary of SPR

**Table S11. Decoy search summary of SPR**

| **Peptide matches** | **in NCBI_ *Nicotiana benthamiana*** | **in Decoy** | **FDR** |
| --- | --- | --- | --- |
| --above identity threshold | 88 | 33 | 37.50% |
| --above identity or homology threshold | 88 | 33 | 37.50% |

## Supplementary figure 6. Secondary mass spectrogram

QUERY corresponds to the Query in the Supplementary table 8.

QUERY-950

QUERY-2285

QUERY-4035 QUERY-4467

QUERY-8132

QUERY-9133

QUERY-10898

QUERY-13492

QUERY-13533

QUERY-13534

**5.4.5 Mascot Search Results**

## Supplementary figure 7. Mascot search result

gi|284810746 corresponds to the Access in the Supplementary table 6.


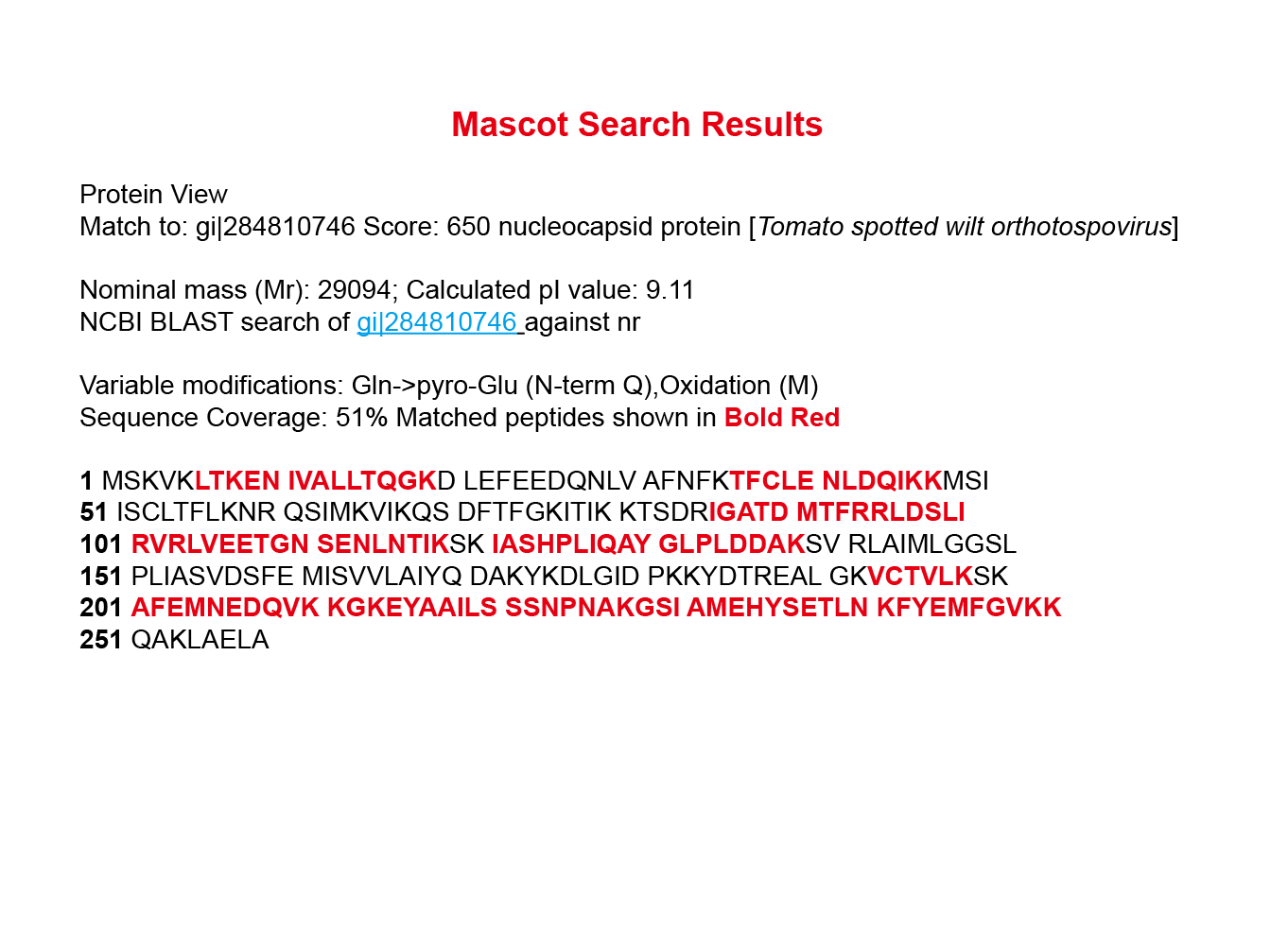
 **Figure S6. Mascot search results** (gi|284810746)

# 7. GST pull down related data

### 7.1. Prokaryotic expression

**7.1.1 Target sequence information**

Name: TSWV N

Expression systems: E.coli Expression System

Gene Sequence:

ATGTCTAAGGTTAAGCTCACTAAGGAAAACATTGTTGCTTTGTTGACACAAGGCAAAGATCTTGAATTTGAAGAAGATCAGAATCTGGTAGCATTTAACTTCAAGACTTTTTGTCTGGAAAACCTTGACCAGATCAAGAAGATGAGCATTATTTCATGTCTGACATTCCTGAAGAATCGTCAGAGTATAATGAAGGTTATTAAGCAAAGTGATTTTACTTTTGGCAAAATCACTATAAAGAAAACTTCAGACAGGATTGGAGCCACTGACATGACCTTCAGAAGGCTTGATAGCTTGATCAGGGTCAGGCTTGTCGAGGAAACTGGGAATTCTGAGAATCTCAATACTATCAAATCTAAGATTGCTTCTCACCCTCTGATTCAAGCCTATGGATTACCTCTTGATGATGCAAAGTCTGTGAGGCTTGCCATAATGCTGGGAGGTAGCTTACCTCTTATTGCTTCAGTTGATAGCTTTGAGATGATCAGTGTTGTCTTGGCTATATATCAGGATGCAAAATACAAAGACCTCGGGATCGATCCAAAGAAGTATGACACCAGGGAAGCCTTAGGGAAAGTTTGCACTGTGCTAAAAAGCAAAGCATTTGAAATGAATGAAGATCAGGTGAAGAAAGGGAAAGAGTATGCTGCTATACTTAGCTCCAGCAATCCTAATGCTAAAGGAAGTATTGCTATGGAACATTACAGTGAAACTCTTAACAAGTTCTATGAAATGTTCGGGGTTAAAAAACAGGCAAAACTTGCAGAACTTGCTTAA

Amino acid sequence:

MSKVKLTKENIVALLTQGKDLEFEEDQNLVAFNFKTFCLENLDQIKKMSIISCLTFLKNRQSIMKVIKQSDFTFGKITIKKTSDRIGATDMTFRRLDSLIRVRLVEETGNSENLNTIKSKIASHPLIQAYGLPLDDAKSVRLAIMLGGSLPLIASVDSFEMISVVLAIYQDAKYKDLGIDPKKYDTREALGKVCTVLKSKAFEMNEDQVKKGKEYAAILSSSNPNAKGSIAMEHYSETLNKFYEMFGVKKQAKLAELA-*

**7.1.3 Expression vector**

The cDNA sequence was cloned in PEGX6p-1 expression vector.

Cloning strategy: BamHI/XhoI

**>** **TSWV N (258AAs; 28.89KDa)**

MSPILGYWKIKGLVQPTRLLLEYLEEKYEEHLYERDEGDKWRNKKFELGLEFPNLPYYIDGDVKLTQSMAIIRYIADKHNMLGGCPKERAEISMLEGAVLDIRYGVSRIAYSKDFETLKVDFLSKLPEMLKMFEDRLCHKTYLNGDHVTHPDFMLYDALDVVLYMDPMCLDAFPKLVCFKKRIEAIPQIDKYLKSSKYIAWPLQGWQATFGGGDHPPKSDLEVLFQGPLGSMSKVKLTKENIVALLTQGKDLEFEEDQNLVAFNFKTFCLENLDQIKKMSIISCLTFLKNRQSIMKVIKQSDFTFGKITIKKTSDRIGATDMTFRRLDSLIRVRLVEETGNSENLNTIKSKIASHPLIQAYGLPLDDAKSVRLAIMLGGSLPLIASVDSFEMISVVLAIYQDAKYKDLGIDPKKYDTREALGKVCTVLKSKAFEMNEDQVKKGKEYAAILSSSNPNAKGSIAMEHYSETLNKFYEMFGVKKQAKLAELA*

Features: GST tag：[1-220]；

PreScission site

Thrombin site [228-229]；

# 8. Amino acid sequence alignment of "hemocyanin subunit 1 precursor"

### 8.1. Amino acid sequence alignment

## Supplementary figure 8. Amino acid sequence alignment of "hemocyanin subunit 1 precursor"


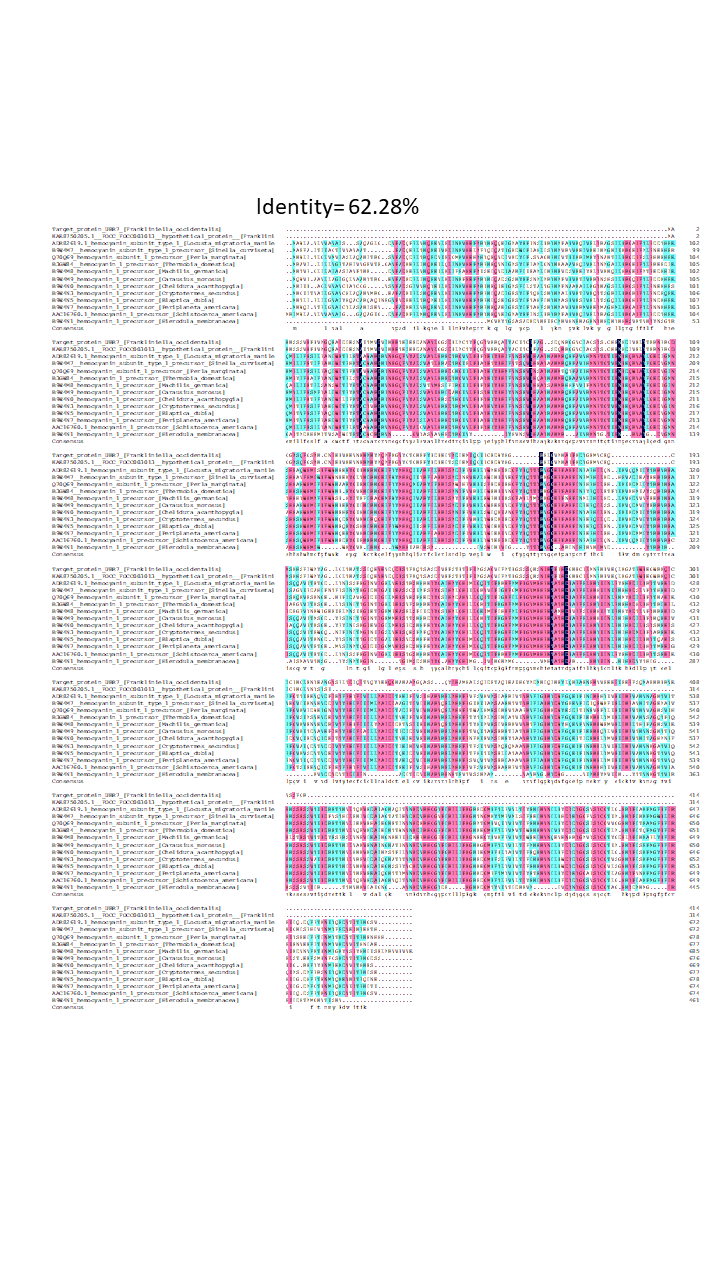


**Figure S8. Amino acid sequence alignment of "hemocyanin subunit 1 precursor"**

### 8.2 Amino acid sequence information for “hemocyanin subunit type 1 precursor” sequence alignment

>Target protein UBR7 [Frankliniella occidentalis]

MAEKSSSVEPPVNGQEADDDESNAITMVDVLKEETELEEDANAVLGGSDPLNCTYPQGYVKRQALYACITCIPAGSDQNRGGVCLACSYSCHENHDLVELYTKRNFRCDCGNSQFGSNKCNLEPVKEVNEKNKYNQNFKGVYCTCKRPYPDPEDTNDDEMIQCIICEDWYHGRHLGVNKAIPKDYGEMVCEQCMSKHSFLWNYAGLCLTKATSSDQEVEVDQDPSTFKQTSASDDVEPSTPTIPINGSAGVDFNTPGSSSQKSNIETPIHDGKECILMNFKPVEQLKGATFWPEGWRKQLCLCIKCLENYEANGVSFLTDLQDTVQYYEEQGKAKAANGQASSQYDHAMHALSQLDRTAQIEAIHGYNDMKDQLKEYLQKFAENRKVVREEDIREFFSQMARKRPRVEVSSFCR

>KAE8750205.1 (FOCC_FOCC003013) hypothetical protein [Frankliniella occidentalis]

MAEKSSSVEPPVNGQEADDDESNAITMVDVLKEETELEEDANAVLGGSDPLNCTYPQGYVKRQALYACITCIPAGSDQKRGGVCLACSYSCHENHDLVELYTKRNFRCDCGNSQFGSNKCNLEPVKEVNEKNKYNQNFKGVYCTCKRPYPDPEDTSDDEMIQCIICEDWYHGRHLGVNKAIPKDYGEMVCEQCMSKHSFLWNYAGLCLTKATSSDQEVEVDQDPSTFKQTSASDDVEPSTPTIPINGSAGVDFNTPGSSSQKSNIETPIHDGKECILMNFKPVEQLKGATFWPEGWRKQLCLCIKCLVNYSISI

>ADR82619.1 hemocyanin subunit type 1 [Locusta migratoria manilensis]

MAWLAVLVVAVALSSAQAGLGDVPADQPFLVKQREVLRLLNKVHEPNRYKEQQELGNAYEPINSLPRYKNPAPVKQLVRLYRAGSLLPRGAIFTLFDDTHREQMILLFESLLFANDWETFLRTAAWARDRVNEGQFVYALSVAVLHREDTRGVVLPPPYEIYPHLFVNSEVIHAAYKAKMRQEPAVVHMNFTGTIRNPEQRVAYLGEDLGMNSHHAQWHMDSPFWWKEEEYGIPKERKGELFYYMHHQLIARFDLERLSNDLPFVEPLYWNERIKDGFYPQTTYRVGGEFPARPDNFAFHDLQNIKVQQMIDYTRRVREAISQQAVITETGDLYNISSPEGINVLGELVEPSTRSKHREYYGALHNYGHIMLGQITDPKRKFNMPPGVMEHFETATRDPAFFRLHKYIDNLFYEHKDLLPRYTVEELDLPGTTIEEVQVDPLETFFEYFDVDLLNALDDTEELPDVSIHARVRRLNHKPFVFSVKVNSDAERLVTVRVFLGPKYDWFGQEIPINDKRHYIVEIDKFVAKVNAGKTVITRKSSESSVTIPDRETTKVLTQKVEDAIAGKAQLTVNKDVRHCGYPDRLLLPKGRRDGMPFTLFVVLTDYEKDKVNDLPYDYDYGGSVSYCGTLGHRYPDAKPMGFPFDRRIDQDQFFTKNIYQRDVTITFKGSV

>B9W4M7 hemocyanin subunit type 1 precursor [Sinella curviseta]

MARFALTLIACTLVVAVAATPADQEFLTKQKEIVKLLNKVHELNFYQDQATIGKDWDPLAHLDSYKNVRVVKELVKELKNGKLIKRGEIFNLFNEEHRREMILLFETLFFAKDWDTFHKTACWARDKINEGQFVYALSVATLHRADTRGIRLPPAYETYPHLFVTSQVIHEAYAAKMRQEPAVIHMNFTGTVRNPEQRVAYFGEDVGMNSHHAVFHMDWPFWWNEEKYGLTKDRKGELFWYMHHQLITRFDAERLSNDLNEVEALHWDKPIVEGFYPQTTYRKGGEFPARPDNFMFHDLKDHRVADLEAYEERIREAISAGVIFDAHDPNTFISLNNTEGIDKLGAIIEASSCSINPSFYGSLHNLGHIILGRVVDPLGKFGMPPGVMEHFETATRDPAFFRLHKHIDNIFKEHKDSLVPYTHEELDVEGVDIKNVEVDDLVTYFEDFDIDMLNALDDAAGLTDVDIKARVQRLNHKPFGIKIIANSAAEKTVTVRLFLAPKYDWYGREVPLDIQRWKFIELDKFAVKLTAGENAIVRKSSESSVTIPDPVSTHDLRKLVDDAIAGTATLEVDKDVRHCGVPDRLLLPKGKTNGMKYTMFVMLSDFEEDKVNDLPHDYEYGGFVSYCGTINHKYPDKKPMGWPLDRKICKDSFHDVTNMYFRDVEIKFEHTH

>Q70Q69 hemocyanin subunit 1 precursor [Perla marginata]

MKWLLTLGLVMVLASLAQAKLTRGSVPADQDFLTRQRDVIRLCMKVHEHNHYQEQVDLVKDYDPSVAGKFKDVTPIKRLMKYYNAKTLLPRGDIFSLFHKEHREEMILLFESFLFAQDWDTFFKTAVWARDRINEGQFVYALSVAVLHREDCKGIILPPAYEIYPHMFVNSEVINSAYKAKMTQTPAIIHMNFTGTIRNPDQWIAYLGEDVGLNSHHAHWHMDFPFWWKAAEYGIEKDRKGELFYYMHHQMIARYDFERLSNWLHFVEPISFEDEIEHGFYPQTTYRVGGEFPARPDNFHFHDLEHIKIKDMLDYTRRIKEAISKQKVRSKNGEKIPLDAVHGIDILGDLMEPSVESPHEDYYGSLHNDAHVLLGQITDPLGKFDLPPGVMEHFETATRDPAFFRLHKHIDNLFKMYKDLLPPYTKAELEFPGVKVLDWEIGNLVTYFEDFDIDMLNALDDTADLPDVDVKARVQRLNHEPFTWALHMESDKEVTAAFRVFLGPKKDWYESDFTINEVRPYLIEIDKFVTKVVAGKSVIHRKSSESSVTIPDRETTKVLLEKVEHALEGKETLNVNKDERHCGYPDRLLLPKGRNTGMPVQIYVIVTDFEKEKVNDLPYDYDYGGSLSYCGVVGGHKYPDTKAMGFPFDRRIYSREDFFTDNMYTKDVTITFKENHHH

>B3GW84 hemocyanin 1 precursor [Thermobia domestica]

MRAVLLLLLVGFTAHLVVGRVTRGAVPADKEFLLKQKEILQLLNKVHEPNRFKEQVNLGNSYDPLANLGNYKKAAAVHQLVKLYNNGALLPRGEIFTLFRERDLEEMIYLFEALFYANDWNTFLSTACWARDRINEGQFVYALSVAVLHREDTKGVVLPPAYEIYPHLFVNSEVIHQAYKAKMRQEAAVVRMNFTGTIRNPEQRVAYFGEDIGMNSHHSHWHMDFPFWWKPEYGVEKDRKGELFYYMHHQLIARFDLERLSNNLPVVRPLDWEKPIVNGFYPQTTYRKGGEFPARPDNFYFQDLETPYIKVKHMIAYSQRIREAIARGVVLTKSGELYSINDTTGINTLGELIEPSVFSRHREYYGALHNYGHILLGRITDPKGKFNMPPGVMEHFETATRDPAFFRLHKYIDNLFKEHKDHLPHYTRDELLLPGVSINSVEVDELVTYFEDFDIDLLNALDDTVELPDVEIKARVRRLNHRPFTYTINLNSDKDATVIVRVFLGPKYDWFGQEIPLEKKRLYMIELDKFVAKVSAGQTPIQRKSSESSVTIPDRETTKVLVQRVKDALEDKTTWNVNKDLRHCGFPDRLLLPKGKKEGMPFTLFVMVTDWEKEKVNDVPYDYDYGGSISYCGTLNHKYPDTQPMGYPFDRRIENVEEFLTPNMYVKDVVITHNDAE

>B9W4M8 hemocyanin 1 precursor [Machilis germanica]

MKTVFCLLLALAASLAVPTKKDVPADHEFLVKQREILKLLFRAHEPNISEDQVDIAAKFDIEANLDKFKEVDSVKELYELYVKKQLLHRGEIFNTFEDKHLEQAILLIETLLSANSWDTLYRTACWARDKVNEGQFVYALSVTVMHSDFLRGIILPPPYEIYPHLFVNSEVIHEAYSAKMRHHPVVIPMNFTGTIRNPEQRVAYFGEDIGLNTHHHYWHMNFPFWWSPHYDTKFDRAGEMFWYMHHQLVARYDLERLSNYLPEVEPLEWFKPIKSGFAPLTMYRKGGEFPNRPDNMLLHDLHDLKVHDVVVFEERIKEAIDRGFVYNKWIGEKIRLNNSIEGIETLGRMIEASRLSPDIDYYGSIHNLGHILLGEIMDPDHKFNLPPGVMEHFETAMRDPVFFSLHKHIDYIFKHYKDTLVPYKREELDFPGVKVENVEVDRLVTFFEPFDIDLYNALDDDKTVSDIEIKARVQRINHKPFTYHINVESDVERKVVVRTFIGPKYDWYHQEVPVNEKRWEMVELDKFLTHIPAGKSVIERLSTESTVTTPDYESFRSLVNRVDEALKGNKEFIIDEEFRHCGLPDRLLIPKGNEEGYPVKFFVIVTDWEEDKVNQEVENHRYGNVYSYCGTYGDRLYPDKKAFLYPFDRVIKDVNVFKTPNMFGKYVSIYHKDISELNRVVPVVEE

>B9W4M9 hemocyanin 1 precursor [Carausius morosus]

MQWVIAAVLLAGLGQLVAAKYTRGEVPADKDFLLKQREVLKLLNKPHEPNRYKDQADSGSKYEPSNNLNRYKNPVPVKTLVKRYNSHSLLPRGQIFTLFDDKHREEMVLLFESMFYALDWDTFYKTACWARDRINEGQFIYALSVAVIHRDDAKGIVLPPSYEIYPHLYVNSEVIHAAYKAKMRQEPAVVRMNFTGTIRNPEQRVAYLGEDLGMNSHHAHWHMDFPFWWKPEEYGIDKDRKGELFYYMHHQLIARFDLERLSNDLPPVEPLGWRERIVDGFYPQTTYRVGGEFPARPDDFEFQDLSSIKVQDMVDYERRVREAISQQAVITMSGDYFSLNDTTGINTLGEMMEPSTTSKHRDYYGALHNYGHILLGKITDPKGKFNMPPGVMEHFETATRDPAFFRLHKHIDNLFKLHKDLLPPYKQEELVLPGVKITDVAVEPLETYFEDFDADLLNALDDTIDLDDVEIKARVRRLNHKPFTVQITAESERETLATVRIFLGPKYDWFGQEIPLEEKRQYLVEIDKFVTKVNSGKTTIQRKSSESSVTIPDRETTKLLVAKVENAINGKATINVNKDVRHCGYPDRLLLPKGKKGGMPFTLYVILTDFNKEKVNDLPYDYDYGGSLSYCGTINHKYPDSRPMGFPFDRRLSTEEFSMPNFCSKDVTITFKGDSS

>B9W4N0 hemocyanin 1 precursor [Chelidura acanthopygia]

MKLIPACLVVAVCIAYCCGVSVPADSGLVVRQYEILKLLNKVHEPNRIKEQIEIGNSFDLSTALTGFKNPNAAKALLKGYKAGSLLPRGSIFTLFNEKHRSEMILLFETFFYANDWDLLFKTACWARDRINEGQFVYALSVAVLHRSDTHGIVLPPPYEIYPHLFVNSEVIHAAYKAKMRQEPAIVRMNFTGTIKNPEQRVAYLGEDIGMNAHHAHWHMDFPFWWKEHEYGIHKDRKGELFYYMHHQLIARFDLERLSNNLPFVEPLSWDQKIANGFYPQTTYRVGGEFPARPDNYAFHDLENIKIKDMIDYTRRIREAIAQQAVITKSGEYFYLNDSKGIEVLGDLMEPSFDSKHPEYYGALHNYGHILLGQITDPKGKFNMPPGVMEHFETATRDPAFFRLHKYIDNLFKLHKDLLPSYSKEELSLDGVQIEDVQIDELTTYFEDFDIDLLNALDDTVELEDVEIKARVRRLNHKPFNFKIEVNSDKEYTAAVRVYIGPKYDWFGQEITLDEKRLYMVEIDKFVTKLTAGHNNIFRKSSESSVTIPDRETTKVLHKKVKDALKNSTPLLVNKDVRHCGYPGRLLLPKGKIEGMPFPLYAIVTDFEQEKVNDLPFDYDYGGSISYCGTLGHKYPDSKPMGYPFDRPIGREFYYPNMFEKDVVITHKES

>B9W4N1 hemocyanin 1 precursor [Hierodula membranacea]

MGVKYTGSASADKDVKRIRCNKVHNRKAGKNYKHDKYKHRSVKTVKYYNSGTRGAITNDKHRMITVSADWDTRTACWCRDRVNGVYASVAVHRDTRGIVYIYHVNSVIHAAYKAKMRAIVRMNTGTIRNRVAYGDVGMNAHHSHWHMDWWKYGVKDRKGYWMHHIARDRSNDVSWDKIVDGYTTYRVGGARDNDHIKVKDMVDYTRRIRAISMAVVTKSGYYSNNTKGINTGIMSDSKHYYGAHNYGHIMGIVDKGKNMGVMHTATRDARHKYIDNKIHKDVYTHDGKVVDDVDVTYDDIDNADDTDDVIKARVRRNHTRVTVSSKNANVAVRVGKYDWGVINKRYMVIDKYTTVNKGTTVIRKSSSSVTIDRTTKVKKVDAIGNGAVNKDVRHCGYDRKGKKDGMTYVIVTDDKKVNDVDYNYGGSISYCGTAGHKYDNKMGDRRIDDHTNMGKVTISKV

>B9W4N3 hemocyanin 1 precursor [Cryptotermes secundus]

MKCILTVALLGVAVCHLAQAKVMRGEAPADKIFLSKQREVLKLLNKVHEPNRFKDQADLGRSYDPTQHLSKYKNALPVKTLVKQYTSGRLLPRGRIFTLFNDRQREQMITIFESLFYAEDWDTFYRTACYVRDRVNEGQFVYALSVAVLHREDTRGMVLPPAYEVYPHLFVNSDVIHAAYRAKMRQEPAVVRMNFTGTVRNPEQRVAYLGEDVGINSHHSHWHMDFPFWWKQDEYGVKKDRQGELFYYMHHQLIARLDLERLSNDLPFVKPLYWEDKIEDGFYPQTTYRVGGEFPARPDNFEFQDLQDIKVKDMVDYTRRIREAISQQSVITRWGQNLPLNDTMGINILGSLVEPSQESPNPQYYGALHNYGHILLGQITDPKGKFDMPPGVMEHFETATRDPAFFRLHKYIDNLFKLHKDMLPPYAREELEFPGVAIQDVTVDDLVTYFEDFDIDLLNALDDTLELKDVEIKARVRRLNHRPFTFSITVNSNQEQMAAVRIFLGPKFDWFGQEIPINEKRLYVIELDKFVAKVNKGATVIQRKSSESSVAIPDRETTKILVRRVDDALQGKATYTVNKDVRHCGYPERLLLPKGKRDGMPFSLFVILTDFDKEKVNDLPWDYDYGGSISYCGTVSGHKYPDSKPMGFPFDRQINSDNFFRSNIYQKDVVITFKDSE

>B9W4N5 hemocyanin 1 precursor [Blaptica dubia]

MKTLLFLLLGVALYHQACARQAQINRGVPVDREFLTRQNEILHLLNKVHEPNRFKDQSELGRSYDPTAHFDKYKNASPVKSLVKLYTSGQLLPRGHIFTLFNDKHREQMITVFESLFYAQDWDTFYRTACWARDRVNEGQFVYALSVAVLHREDTRGIVLPPAYEIYPHLFVNSEVIHAAYRAKMRQEPAVVRMNFTGTVRNPEQRVAYLGEDVGMNSHHSHWHMDFPFWWKEQEYGSHKDRKGELFYWMHHQLISRFDLERLSNDLPFVEPLYWDEKIVDGFYPQTTYRVGGEFPARPDNFEFQDLKNIKVKDMVDYTRRIREAISQQAVITKNGDYYSLNDTTGIDTLGALIEPSVDSKHPEYYGALHNYGHIMLGQITDPKGKFNMPPGVMEHFETATRDPAFFRLHKYIDNLFKLHKDLLPKYTQAELSFPGVKVSDVTVDELVTYFEDFDIDLLNALDDTQELDDVEIKARVRRLNHKPFTFHITINSELDATAAVRVFLGPKYDWFGQEIPINEKRLYMVEIDKFVAKVNKGTTVVQRKSSESSVTIPDRETTKDLVKKVHDALEGKSSFYVCKDVRHCGYPDRLLLPKGKKEGMPFTLYVIVTDFEKEKVNDLPWDYDYGGSISYCGVLSGHKYPDSKPMGFPFDRHIDGDHFFTENMYQKEVKITFQDNE

>B9W4N7 hemocyanin 1 precursor [Periplaneta americana]

MKWQLVTFLGVALCYLASAKLSEVPADKEFLVRQREVLRLLNKVHEPNRFKEQAELGRSYDPTAHFSKYKNAVPVKRMVKEYMAGHMLPRGAIFTLFNNKHREEMITVFESFFFAEDWDTFYRTACWARDRVNEGQFIYALSLAVLHREDTRGIVLPPAYEIYPHLFVNSEVIHAAYKAKMRQEPAVVRMNFTGTIRNPEQRVAYLGEDVGMNSHHSHWHMDFPFWWKQEEYGVHKDRKGELFYYMHHQLIARFDAERLSNDLPIVEPLYWDEKIVDGFYPQTTYRVGGEFPARPDNFEFHDLEHIKVKDMMDYTRRIREAISQQAVVTRTGEMVSLNNTQGIDILGCMVEPSHDSKHPEYYGALHNYGHIMLGQITDPKGKFNMPPGVMEHFETATRDPAFFRLHKYIDNLFKLHKDLLPHYTMEELGFNGVTIQDLTVDDLVTYFEDFDIDMLNALDDTAELQDVDIKARVRRLNHKPFSVQVTVNSERDAMAVVRIFLAPKYDWFGQEIPINEKRLYMIEIDKFVTKVNKGTTVVQRKSSESSVTIPDRETTKVLTKRVEEALQGKTTYMVNKDVRHCGYPDRLLLPKGKKDGMIFTMYVIVTDYETEKVNDLPYDYEYGGAISYCGTLAGHKYPVNKPMGFPFDRQIDGDNFCTPNMFQKDVIITFKDTI

>AAC16760.1 hemocyanin 1 precursor [Schistocerca americana]

MRLMWLAVLVVAVALGGAQAGIGDVPADKPFLMKQREVLRLFNKVHEPNRYKEQVELGNAYEPLNSLPRYRNPAPVKQLVRLYRAGSLLPRGAIFTLFDDTHREQMILLFESLLYANDWETFLRTAAWARDRVNEGQFVYALCVAVLHREDTRGVVLPPPYEIYPHLFVNSEVIHAAYKAKMRQEPAVVHMNFTGTIRNPEQRVAYLGEDLGMNSHHSQWHMDFPFWWKEDEYGIRKERKGELFYYMHHQLIARFDLERLSNDLPFVEPLYWTERIKDGFYPQTTYRVGGEFPARPDNFAFHDLQNIKVQQMIDYTRRVRECISQQAVITETGDLYNISSPEGINVLGELIEPSTGSKHREYYGALHNYGHIMLGQITDPKRKFNMPPGVMEHFETATRDPAFFRLHKYIDNLFYEHKDLLPRYTSEELELPGTSIEEVQIDPLETFFEYFDVDLLNALDDTEELPDVSIHARVRRLNHKPFVFSVRVNSEAERFVTVRVFLGPKYDWFGQEIPINEKRHYIVEIDKFVAKVNAGKTVIARKSSESSVTIPDRETTKVLTQRVEDAIAGKVQLTVNKDVRHCGYPDRLLLPKGRRDGMPFTLFVVLTDYEKDKVNDLPFDYDYGGSVSYCGTLGHRYPDAKPMGFPFDRRIDQDSFFTKNIYQRDVTITFKGSV
