# Supplementary figures and images for: The effects of the E3 ubiquitin–protein ligase UBR7 of Frankliniella occidentalis on the ability of insects to acquire and transmit TSWV
Source: PeerJ. 2023 May 9;11:e15385. doi: 10.7717/peerj.15385 (PMC10178284; doi:10.7717/peerj.15385)

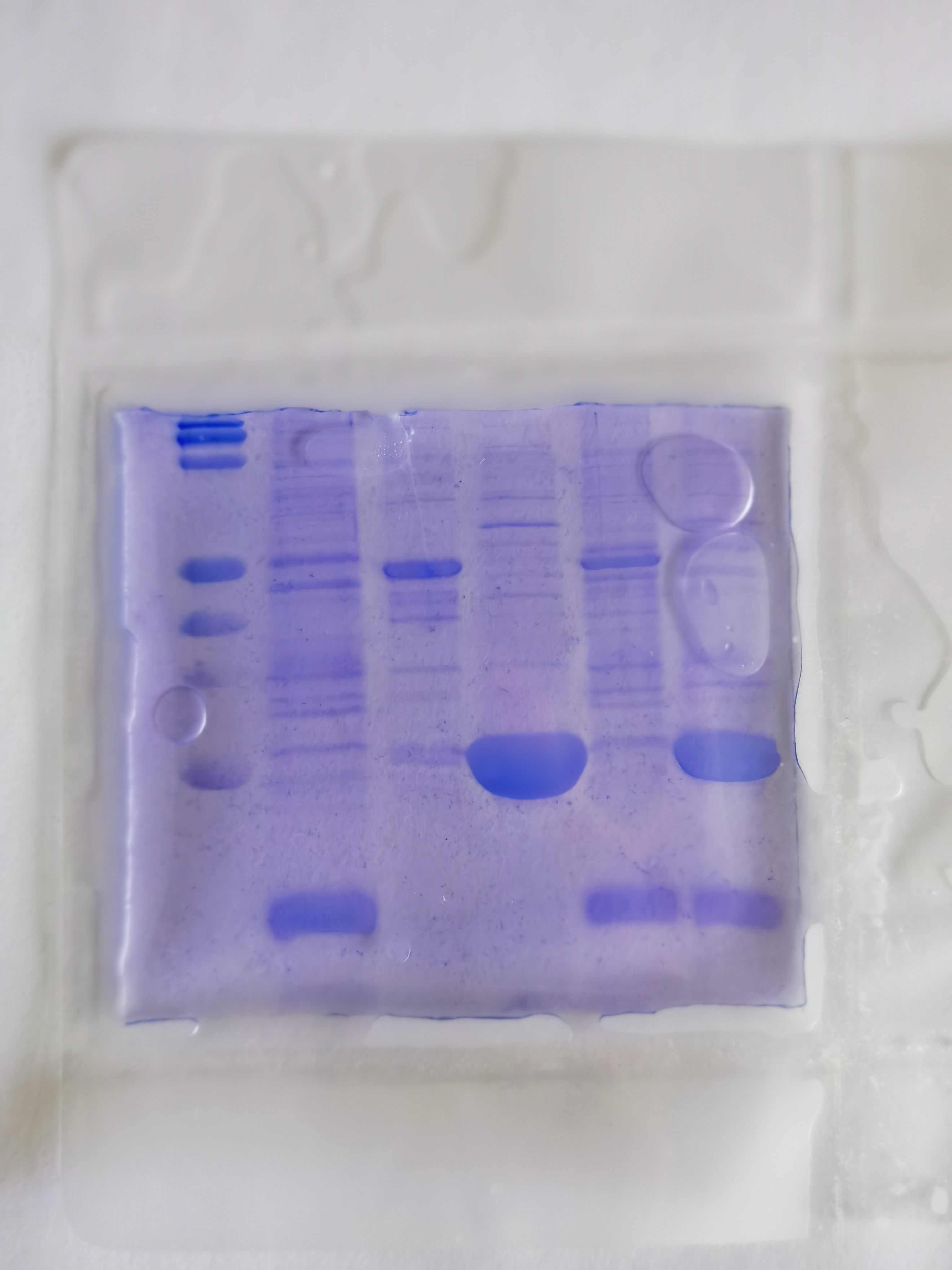

Supplement: Supplemental Information 3 [file peerj-11-15385-s003.zip › Additional file 3/gst pull down and co-IPú¿figure 8ú⌐/figure 8 (a) input-2.png]

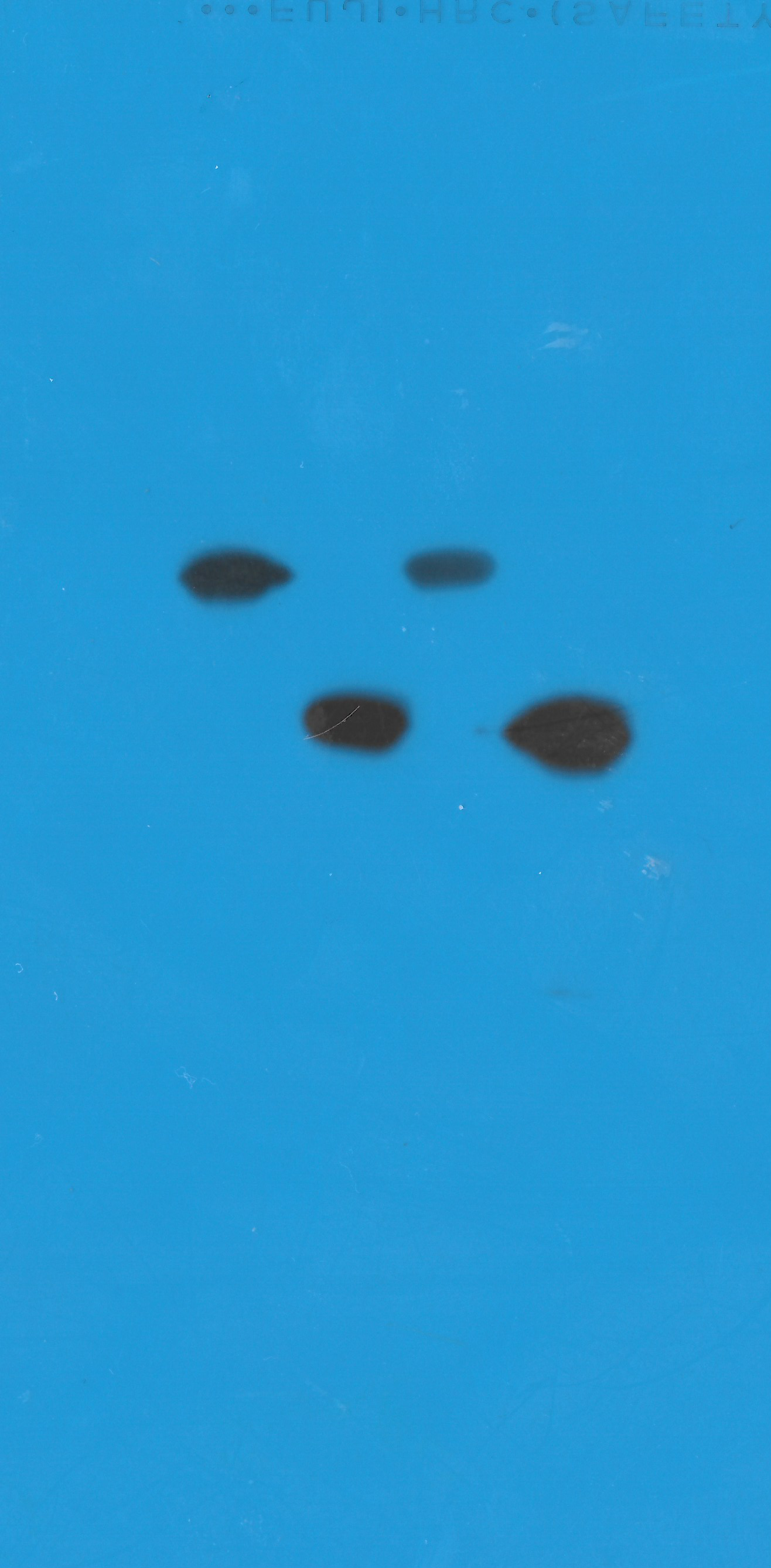

Supplement: Supplemental Information 3 [file peerj-11-15385-s003.zip › Additional file 3/gst pull down and co-IPú¿figure 8ú⌐/figure 8 (a)input anti-GST.png]

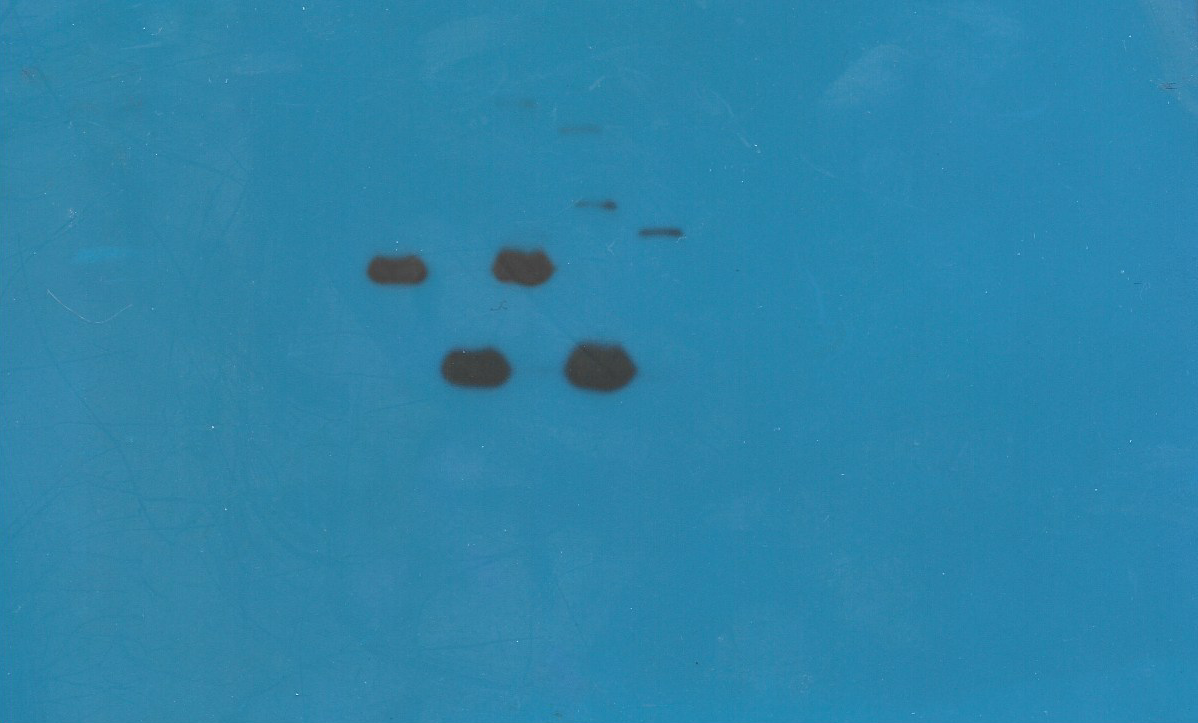

Supplement: Supplemental Information 3 [file peerj-11-15385-s003.zip › Additional file 3/gst pull down and co-IPú¿figure 8ú⌐/figure 8 (b)pull down anti-GST.png]

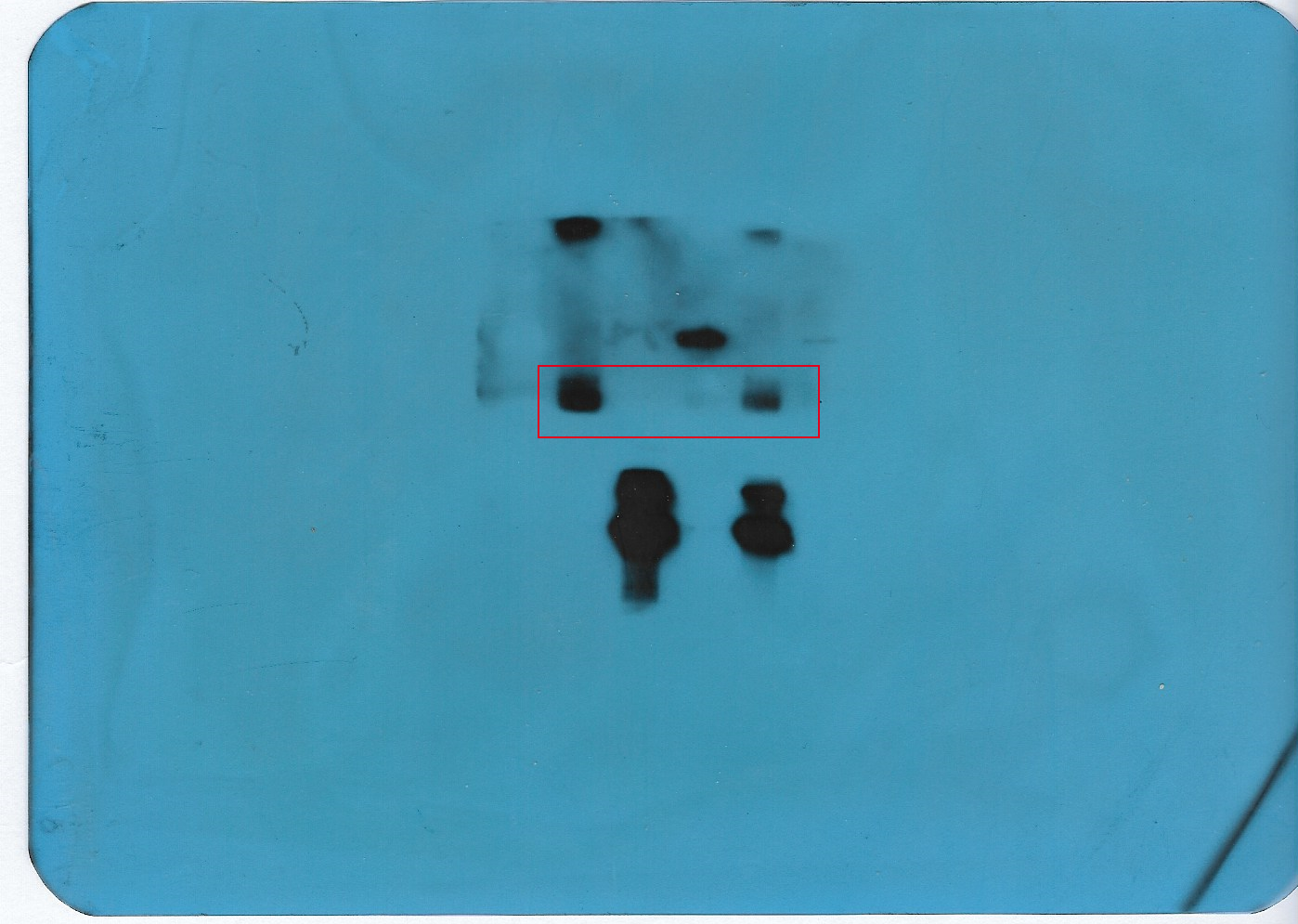

Supplement: Supplemental Information 3 [file peerj-11-15385-s003.zip › Additional file 3/gst pull down and co-IPú¿figure 8ú⌐/figure 8 (b)pull down anti-UBR7.png]

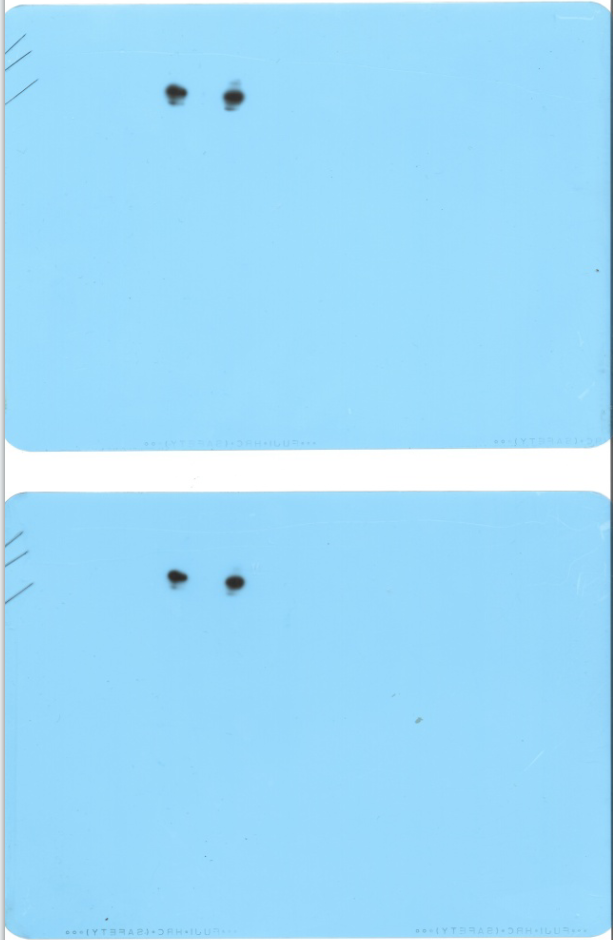

Supplement: Supplemental Information 3 [file peerj-11-15385-s003.zip › Additional file 3/gst pull down and co-IPú¿figure 8ú⌐/figure 8 (c)co-ip anti-TSWV N.png]

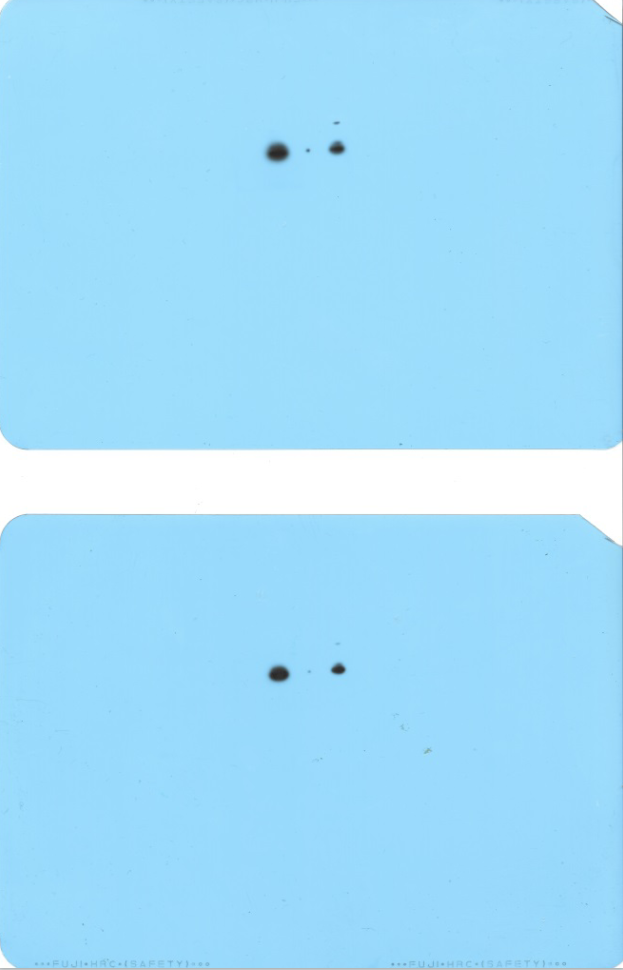

Supplement: Supplemental Information 3 [file peerj-11-15385-s003.zip › Additional file 3/gst pull down and co-IPú¿figure 8ú⌐/figure 8 (c)co-ip anti-UBR7.png]

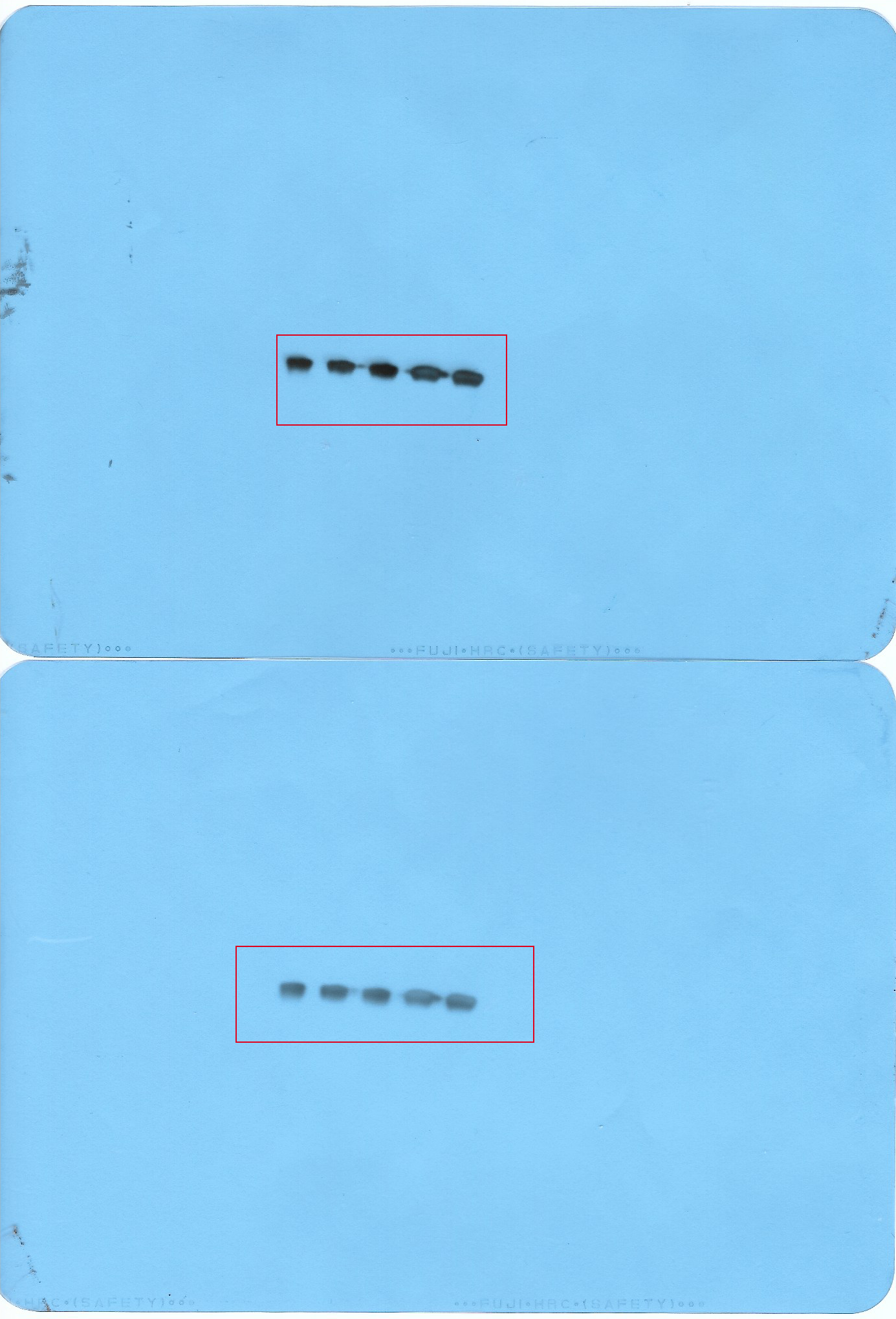

Supplement: Supplemental Information 3 [file peerj-11-15385-s003.zip › Additional file 3/western blot ú¿figure 5ú⌐/figure 5(b) actin.png]

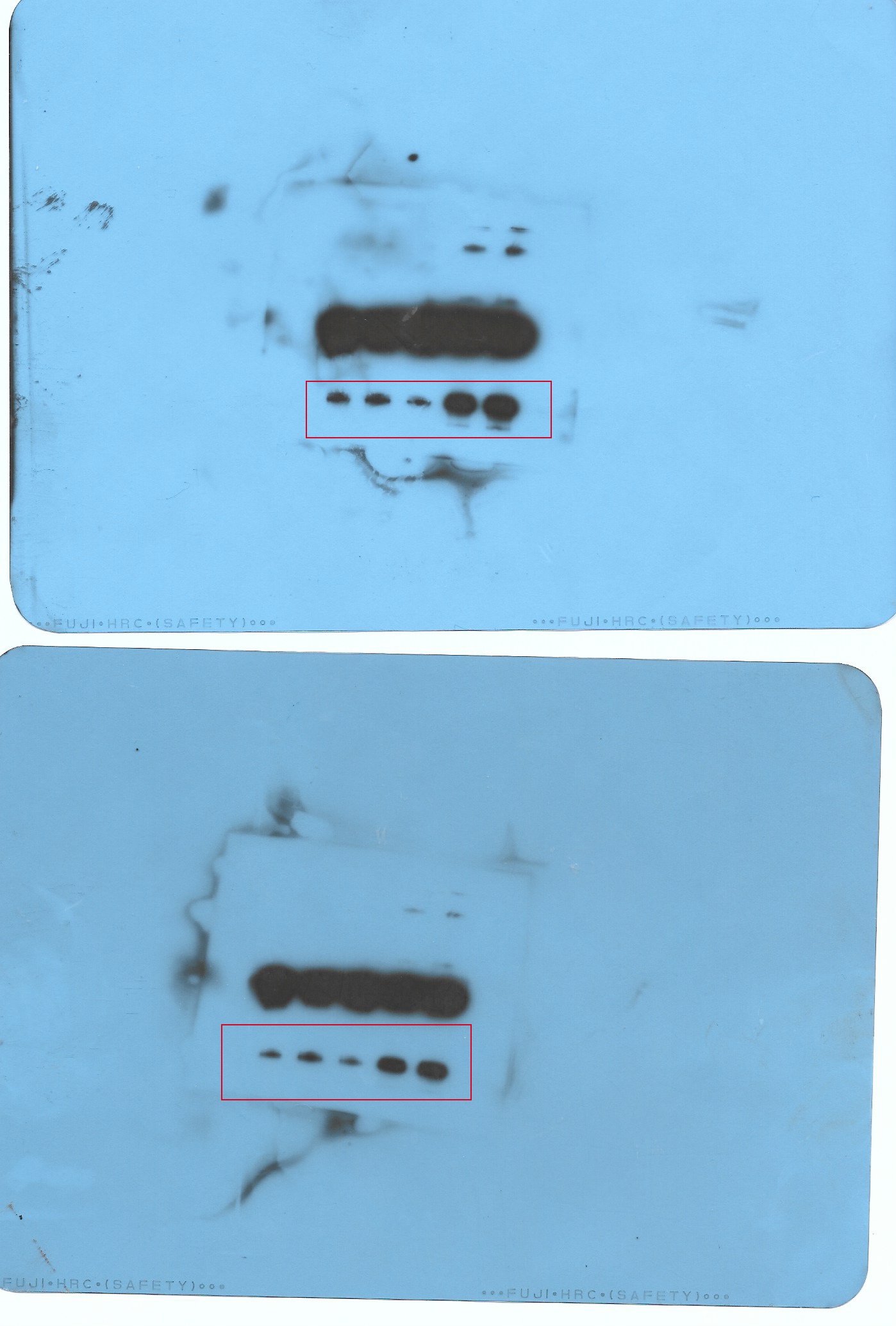

Supplement: Supplemental Information 3 [file peerj-11-15385-s003.zip › Additional file 3/western blot ú¿figure 5ú⌐/figure 5(b) UBR7.png]

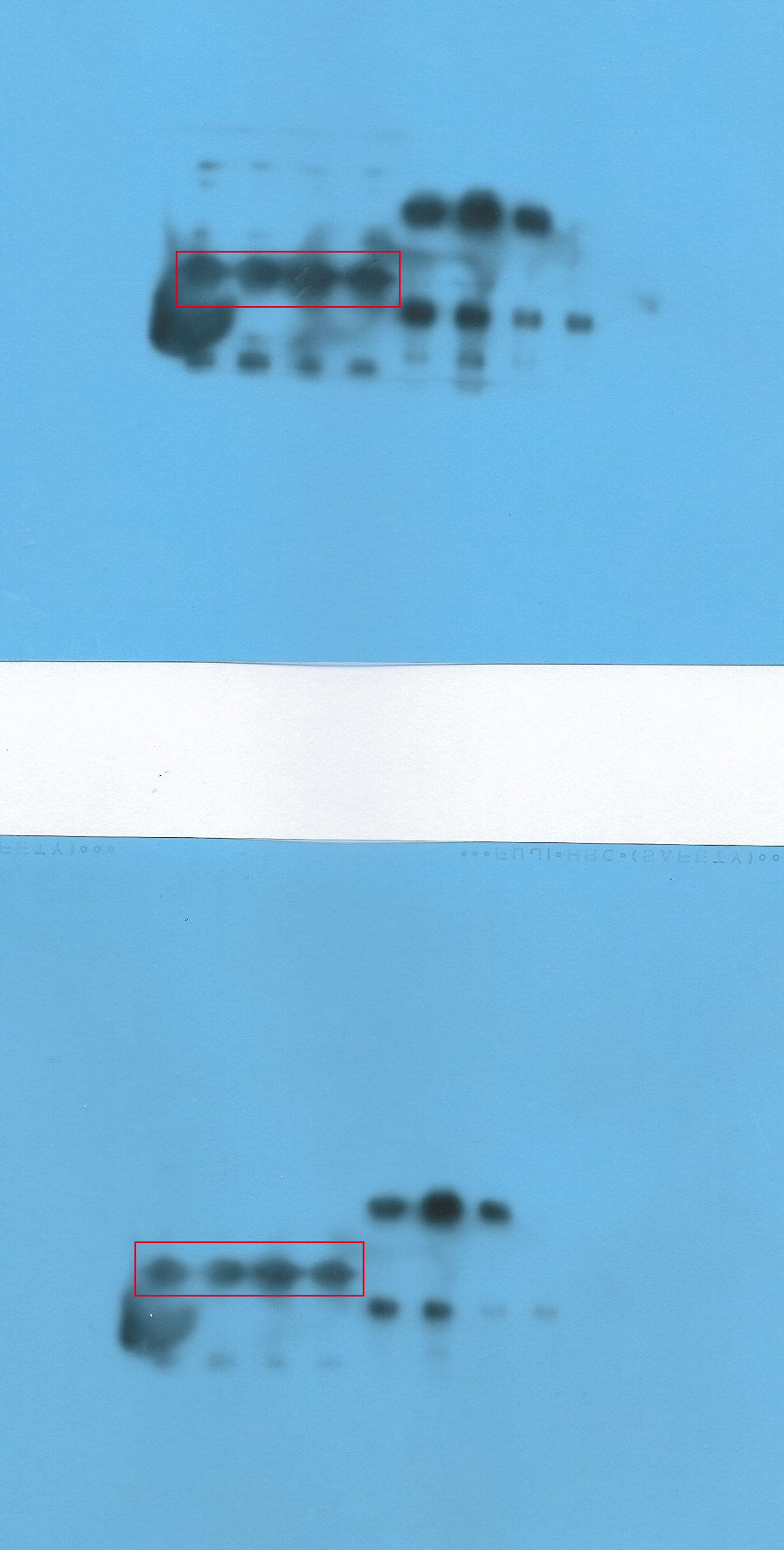

Supplement: Supplemental Information 3 [file peerj-11-15385-s003.zip › Additional file 3/western blot ú¿figure 5ú⌐/figure 5(d) actin.png]

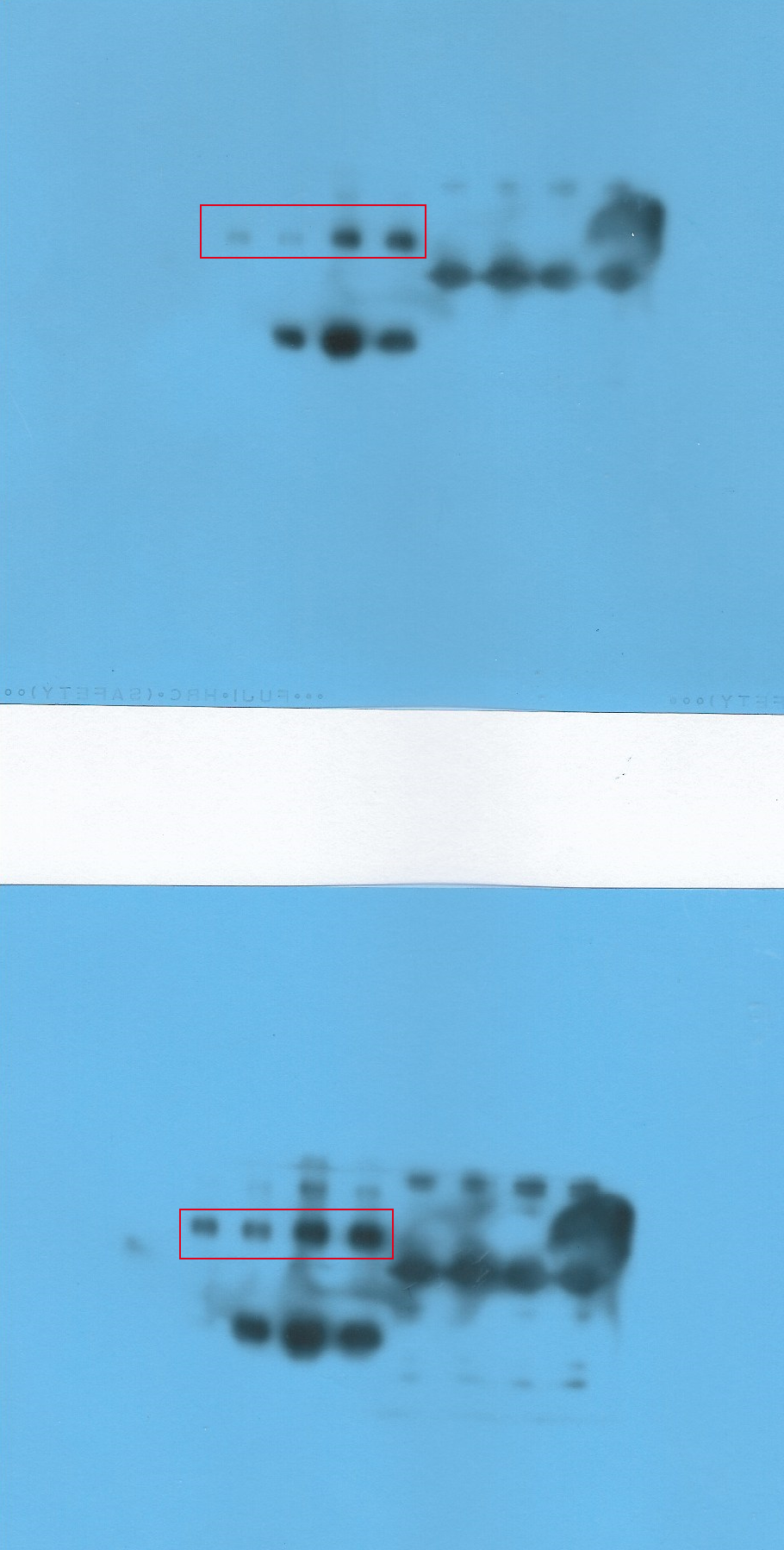

Supplement: Supplemental Information 3 [file peerj-11-15385-s003.zip › Additional file 3/western blot ú¿figure 5ú⌐/figure 5(d) UBR7.png]

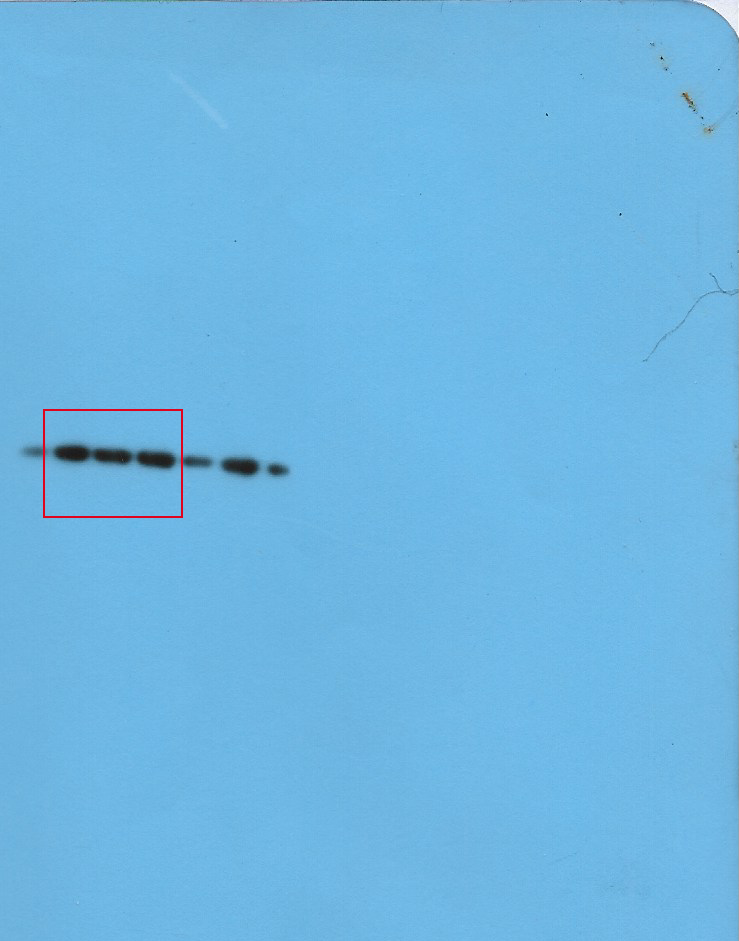

Supplement: Supplemental Information 3 [file peerj-11-15385-s003.zip › Additional file 3/western blot ú¿figure 5ú⌐/figure 5(f) actin.png]

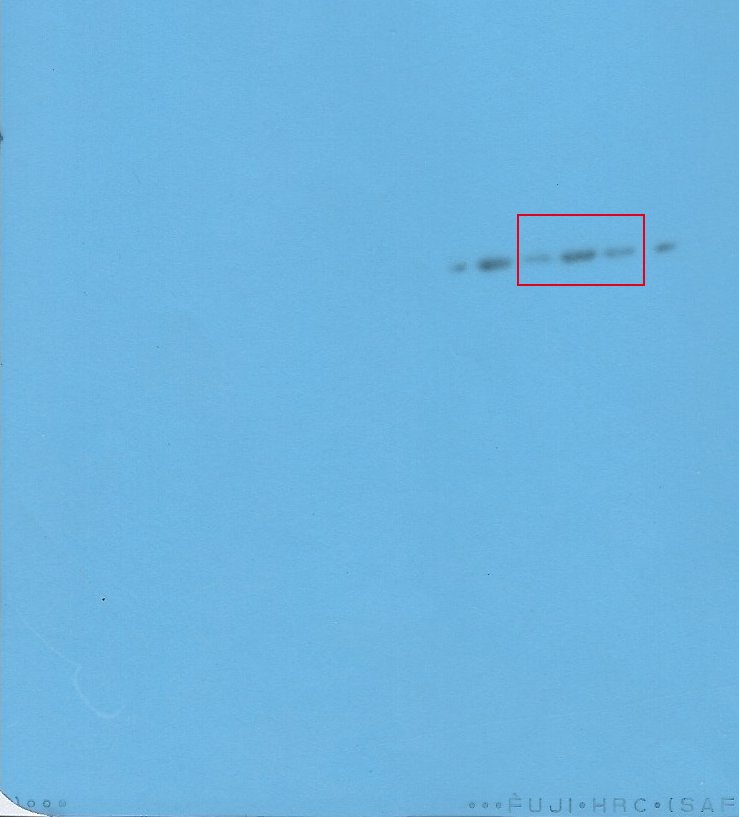

Supplement: Supplemental Information 3 [file peerj-11-15385-s003.zip › Additional file 3/western blot ú¿figure 5ú⌐/figure 5(f) UBR7.png]

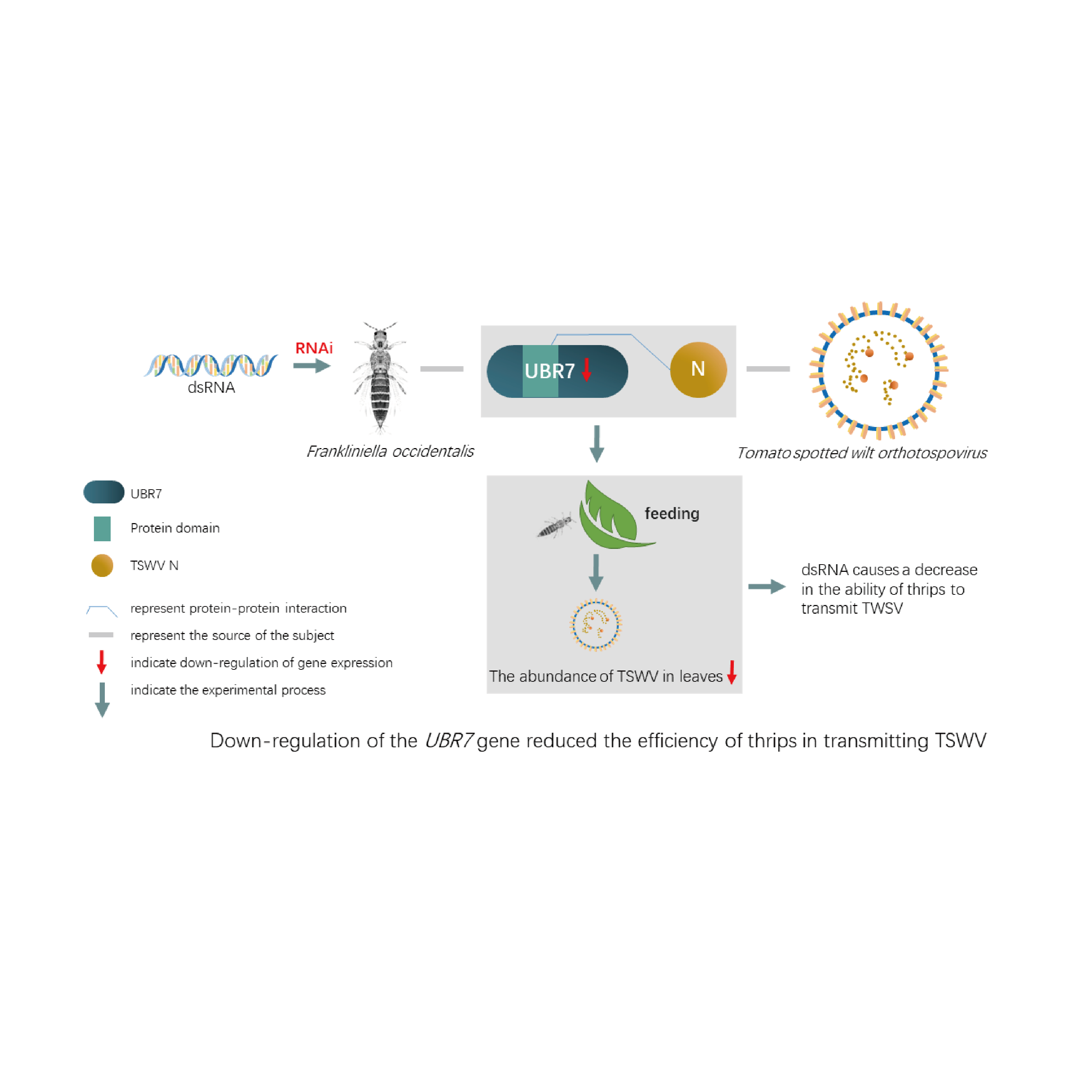

Supplement: Supplemental Information 5 [file peerj-11-15385-s005.png]
