# Supplementary material for: The effects of the E3 ubiquitin–protein ligase UBR7 of Frankliniella occidentalis on the ability of insects to acquire and transmit TSWV
Source: PeerJ. 2023 May 9;11:e15385. doi: 10.7717/peerj.15385 (PMC10178284; doi:10.7717/peerj.15385)
Supplement: Supplemental Information 4 — Each values are representative of data obtained from at least three independent experiments (n ≥ 3). [file peerj-11-15385-s004.docx]

**Experimental records**

**Contents**

[1. Bioinformatic analysis 3](#_Toc121324322)

[1.1 Transcriptome data download information 3](#_Toc121324323)

[1.2 Results of transcriptome analysis 6](#_Toc121324325)

[1.3 Prediction analysis of proteins 7](#_Toc121324327)

[1.4 Homologous alignment of amino acid sequence 9](#_Toc121324329)

[2. Full length verification 10](#_Toc121324331)

[2.1 Sequencing results after TA cloning 10](#_Toc121324332)

[2.2 Putative translated protein sequences 10](#_Toc121324333)

[3. Phylogenetic tree related data 12](#_Toc121324334)

[4. Antibody preparation 20](#_Toc121324336)

[4.1 Project Information 20](#_Toc121324337)

[4.2 Product Information 20](#_Toc121324338)

[4.3 QC (Quality Control) Results 20](#_Toc121324340)

[5. Surface plasmon resonance related data 21](#_Toc121324342)

[5.1. Prokaryotic expression 21](#_Toc121324343)

[5.2 L scale-up purification 23](#_Toc121324346)

[5.3. Surface plasmon resonance 24](#_Toc121324348)

[5.4 Protein mass spectrometry 26](#_Toc121324352)

**Contents**

[Supplementary table 1：Transcriptome information of *Frankliniella occidentalis* from NCBI SPR 3](#_Toc121324603)

[Supplementary table 2：The top twenty most differentially-abundant transcript sequences in three developmental stages of *Frankliniella occidentalis* in response to tomato spotted wilt virus (TSWV) infection 6](#_Toc121324605)

[Supplementary table 3: Bioinformatics prediction analysis of physicochemical properties of proteins 7](#_Toc121324607)

[Supplementary txt 1. Amino-acid sequences used for homologous alignment 9](#_Toc121324609)

[Supplementary txt 2. Amino-acid sequences used for phylogenetic tree 12](#_Toc121324614)

[Supplementary table 4: Information about antibody preparation products 20](#_Toc121324618)

[Supplementary table 5: Quality Control of antibody preparation products 20](#_Toc121324620)

[Supplementary figure 1. Protocol for prokaryotic expression 22](#_Toc121324623)

[Supplementary figure 2. Expression tests of the target protein UBR7-domino 22](#_Toc121324624)

[Supplementary figure 3. Reducing-PAGE analysis. Final sample QC. 2µg of sample loaded 23](#_Toc121324626)

[Supplementary table 6: Experimental parameters of SPR 24](#_Toc121324628)

[Supplementary figure 4. Sample signal diagram 24](#_Toc121324629)

[Supplementary figure 5. Sample elution 25](#_Toc121324630)

[Supplementary table 7: Peptides interacting with UBR7-Domino identified by SPR and LC-MS/MS 26](#_Toc121324632)

[Supplementary table 8: The information of protein mass spectrometry 27](#_Toc121324633)

[Supplementary table 9. Search parameters of SPR 27](#_Toc121324634)

[Supplementary table 10: Decoy search summary of SPR 28](#_Toc121324635)

[Supplementary figure 5. Secondary mass spectrogram 28](#_Toc121324636)

[Supplementary figure 6. Mascot search result 33](#_Toc121324637)

# 1. Figure 1

Figure 1(b)

| The relative abundance of  TSWV in *Frankliniella occidentalis* | | |
| --- | --- | --- |
|  |  |  |
| CK | ds-EGFP | ds-UBR7 |
| 0.384757 | 0.738167 | 0.178256 |
| 0.314693 | 0.647081 | 0.836257 |
| 1.406411 | 0.883939 | 0.529249 |
| 1.051186 | 1.058498 | 1.406411 |
| 1.842952 | 0.493807 | 0.603748 |

Figure 1(d)

|  | The relative absorbance  value in A450 nm | | |
| --- | --- | --- | --- |
|  |  |  |  |
|  | CK | ds-EGFP | ds-UBR7 |
|  | 2.615385 | 2.430769 | 0.123077 |
|  | 5.123077 | 2.507692 | 0.138462 |
|  | 3.692308 | 3.030769 | 0.107692 |
|  | 5.307692 | 2.753846 | 0.046154 |
|  | 4.076923 | 2.307692 | 0.184615 |
|  | 5.030769 | 2.953846 | 0.692308 |
|  | 2.338462 | 3.461538 | 0.676923 |
|  | 2.892308 | 1.892308 | 0.8 |
|  | 3.276923 | 2.138462 | 0.230769 |

# 2. Figure 5

| Figure 5(a) | The relative *UBR7* gene expression  in *Frankliniella occidentalis* | | | | |
| --- | --- | --- | --- | --- | --- |
|  |  |  |  |  |  |
|  | L1s | L2s | Pupea | Female  adults | Male  adults |
|  | 0.525147 | 0.156127 | 0.901747 | 3.865873 | 2.235805 |
|  | 0.341698 | 0.108879 | 0.607432 | 2.991345 | 2.235805 |
|  | 0.463549 | 1.395512 | 0.155049 | 2.849669 | 4.230401 |
|  | 2.363286 | 0.473289 | 0.104443 | 3.075443 | 3.892763 |
|  | 0.24499 | 0.628853 | 0.339338 | 2.568266 | 1.385872 |
|  | 0.111167 | 0.295411 | 1.694423 | 2.267016 | 1.5271 |
|  | 2.950162 | 1.293065 | 1.718077 | 4.726573 | 2.771745 |

| Figure 5(b) | The relative protein expression of *UBR7*  in *Frankliniella occidentalis* | | | | |
| --- | --- | --- | --- | --- | --- |
|  |  |  |  |  |  |
|  | L1s | L2s | Pupae | Female  adults | Male  adults |
|  | 0.846415 | 1.146728 | 0.737097 | 3.739083 | 3.566726 |
|  | 0.937832 | 1.021018 | 0.832213 | 3.559924 | 3.518161 |
|  | 1.215753 | 1.003034 | 0.908529 | 3.686845 | 3.738539 |

| Figure 5(c) | The relative gene expression of *UBR7*  in *Frankliniella occidentalis* | | | |
| --- | --- | --- | --- | --- |
|  | ♀V- | ♂V- | ♀V+ | ♂V+ |
|  | 0.239409 | 1.534266 | 1.811957 | 1.738145 |
|  | 1.678937 | 0.531281 | 4.916216 | 2.281247 |
|  | 1.178987 | 2.293496* | 2.246296 | 1.56548 |
|  | 0.577362 | 0.881204 | 1.523668 | 1.338575 |
|  | 1.354301 | 1.4616 | 1.750235 | 1.284707 |
|  | 0.971003 | 0.655823 | 0.851187 | 1.0226 |

| Figure 5(d) | The relative protein expression of *UBR7*   in *Frankliniella occidentalis* | | | |
| --- | --- | --- | --- | --- |
|  | ♀V- | ♂V- | ♀V+ | ♂V+ |
|  | 1.403776 | 1.555765 | 3.365602 | 3.538077 |
|  | 0.759003 | 0.741723 | 1.649713 | 1.047321 |
|  | 0.837221 | 0.744198 | 2.653087 | 2.772453 |

| Figure 5(e) | The relative gene expression of UBR7  in *Frankliniella occidentalis* | | |
| --- | --- | --- | --- |
|  | Head | Thorax | Abdomen |
|  | 0.696072 | 14.79777 | 4.048278 |
|  | 1.372978 | 15.75031 | 3.573427 |
|  | 0.509554 | 15.2138 | 3.725175 |
|  | 1.421396 | 14.59405 | 3.26551 |

| Figure 5(f) | The relative protein expression of UBR7  in *Frankliniella occidentalis* | | |
| --- | --- | --- | --- |
|  | Head | Thorax | Abdomen |
|  | 1.055116 | 3.460293 | 1.12844 |
|  | 0.921205 | 3.33855 | 0.989763 |
|  | 1.02368 | 2.636569 | 1.237866 |

# 3. Figure 6

| Figure 6(a) | The relative gene expression of UBR7  in *Frankliniella occidentalis* | | |
| --- | --- | --- | --- |
|  |  |  |  |
|  | CK | ds-EGFP | ds-UBR7 |
|  | 0.415524 | 0.797194 | 1.14314 |
|  | 0.797194 | 1.436943 | 0.326013 |
|  | 0.312733 | 0.587639 | 0.448029 |
|  | 0.73867 | 0.559807 | 0.464259 |
|  | 1.322257 | 0.76472 | 0.145895 |
|  | 1.175279 | 1.427017 | 0.554623 |
|  | 1.639212 | 1.2861 | 0.212127 |
|  | 1.599131 | 0.808322 | 0.436943 |

| Figure 6(b) | The survival rate  of *Frankliniella occidentalis* (%) | | |
| --- | --- | --- | --- |
|  |  |  |  |
|  | CK | ds-EGFP | ds-UBR7 |
|  | 0.888888889 | 0.875 | 0.8 |
|  | 0.833333333 | 0.83333 | 0.761904762 |
|  | 0.941176471 | 0.89474 | 0.772727273 |

# 4. Figure 7

Figure 7(b)

| The relative gene expression of UBR7 *in Frankliniella occidentalis* | | | | | | | | | |
| --- | --- | --- | --- | --- | --- | --- | --- | --- | --- |
|  |  |  |  |  |  |  |  |  |  |
| Time | CK | | | ds-EGFP | | | ds-UBR7 | | |
| 0h | 0.809176 | 1.038517 | 1.152307 | 1.152307 | 1.351466 | 0.765527 | 0.91037 | 1.03852 | 0.781612 |
| 6h | 1.003141 | 0.814804 | 0.904082 | 1.120797 | 0.814804 | 1.113055 | 0.51211 | 0.75499 | 0.69473 |
| 12h | 0.996212 | 0.891635 | 1.176519 | 1.003141 | 0.975711 | 0.989331 | 0.448918 | 0.53757 | 0.519259 |
| 24h | 0.935964 | 1.082619 | 1.296413 | 0.826178 | 1.428524 | 0.867253 | 0.124527 | 0.19951 | 0.266928 |
| 48h | 0.989331 | 0.843538 | 1.02422 | 1.176519 | 1.003141 | 1.07514 | 0.340217 | 0.45519 | 0.415962 |
| 72h | 0.754988 | 1.017145 | 1.176519 | 1.243602 | 1.010119 | 1.097731 | 0.533857 | 0.98933 | 0.749773 |
| 96h | 0.809176 | 1.060339 | 1.33286 | 0.814804 | 0.760239 | 1.33286 | 1.176519 | 1.26096 | 0.609005 |

Figure 7(c)

| The relative abundance of TSWV in *Frankliniella occidentalis* | | | | | | | | | |
| --- | --- | --- | --- | --- | --- | --- | --- | --- | --- |
|  |  |  |  |  |  |  |  |  |  |
| Time | CK | | | ds-EGFP | | | ds-UBR7 | | |
| 0h | 0.909261 | 1.001919 | 1.08882 | 1.158908 | 1.216525 | 1.044466 | 0.80819 | 0.83091 | 1.199777 |
| 6h | 0.915585 | 0.80819 | 1.081299 | 1.135058 | 0.713391 | 0.860213 | 0.9813 | 0.92195 | 0.896743 |
| 12h | 0.896743 | 1.224987 | 1.32204 | 0.915585 | 1.066413 | 0.884397 | 0.69871 | 0.94133 | 0.621043 |
| 24h | 0.791557 | 1.191489 | 1.250726 | 0.764594 | 1.015905 | 1.030087 | 0.54441 | 0.41259 | 0.406906 |
| 48h | 1.312908 | 0.954466 | 0.813811 | 0.830911 | 1.10402 | 0.775267 | 0.612493 | 0.45779 | 0.487261 |
| 72h | 1.378181 | 0.733448 | 1.285889 | 0.754068 | 1.208122 | 1.135058 | 0.674909 | 0.78066 | 0.764594 |
| 96h | 0.802607 | 0.90298 | 1.166969 | 1.158908 | 0.890548 | 1.191489 | 0.878288 | 0.95447 | 0.522233 |
